# *Thermobifida fusca* Cel6B moves bidirectionally while processively degrading cellulose

**DOI:** 10.1186/s13068-024-02588-0

**Published:** 2024-12-04

**Authors:** Madeline M. Johnson, Antonio DeChellis, Bhargava Nemmaru, Shishir P. S. Chundawat, Matthew J. Lang

**Affiliations:** 1 Department of Chemical and Biomolecular Engineering, Vanderbilt University, Nashville, TN 37235 USA; 2 Department of Chemical and Biochemical Engineering, Rutgers, The State University of New Jersey, 98 Brett Road, Piscataway, NJ 08854 USA; 3 Department of Molecular Physiology and Biophysics, Vanderbilt University School of Medicine, Nashville, TN 37232 USA

**Keywords:** Cellulase, Cellobiohydrolase, Cellulose, Optical tweezers, Biofuels, Carbohydrate-binding module, Biofuel, *Thermobifida fusca*, Cel6B

## Abstract

**Background:**

Cellulose, an abundant biopolymer, has great potential to be utilized as a renewable fuel feedstock through its enzymatic degradation into soluble sugars followed by sugar fermentation into liquid biofuels. However, crystalline cellulose is highly resistant to hydrolysis, thus industrial-scale production of cellulosic biofuels has been cost-prohibitive to date. Mechanistic studies of enzymes that break down cellulose, called cellulases, are necessary to improve and adapt such biocatalysts for implementation in biofuel production processes. *Thermobifida fusca* Cel6B (*Tf*Cel6B) is a promising candidate for industrial use due to its thermostability and insensitivity to pH changes. However, mechanistic studies probing *Tf*Cel6B hydrolytic activity have been limited to ensemble-scale measurements.

**Results:**

We utilized optical tweezers to perform single-molecule, nanometer-scale measurements of enzyme displacement during cellulose hydrolysis by *Tf*Cel6B. Records featured forward motility on the order of 0.17 nm s^−1^ interrupted by backward motions and long pauses. Processive run lengths were on the order of 5 nm in both forward and backward directions. Motility records also showed rapid bidirectional displacements greater than 5 nm. Single-enzyme velocity and bulk ensemble activity were assayed on multiple crystalline cellulose allomorphs revealing that the degree of crystallinity and hydrogen bonding have disparate effects on the single-molecule level compared to the bulk scale. Additionally, we isolated and monitored the catalytic domain of *Tf*Cel6B and observed a reduction in velocity compared to the full-length enzyme that includes the carbohydrate-binding module. Applied force has little impact on enzyme velocity yet it readily facilitates dissociation from cellulose. Preliminary measurements at elevated temperatures indicated enzyme velocity strongly increases with temperature.

**Conclusions:**

The unexpected motility patterns of *Tf*Cel6B are likely due to previously unknown mechanisms of processive cellulase motility implicating irregularities in cellulose substrate ultrastructure. While *Tf*Cel6B is processive, it has low motility at room temperature. Factors that most dramatically impact enzyme velocity are temperature and the presence of its native carbohydrate-binding module and linker. In contrast, substrate ultrastructure and applied force did not greatly impact velocity. These findings motivate further study of *Tf*Cel6B for its engineering and potential implementation in industrial processes.

**Supplementary Information:**

The online version contains supplementary material available at 10.1186/s13068-024-02588-0.

## Background

Cellulose is a polymer consisting of repeating glucose units that is largely responsible for the structural integrity of plant and algal cell walls. It is often found as both ordered highly crystalline bundles and disordered amorphous entanglements of multiple polymer chains, even within the same microcrystal network. Cellulosic materials, if degraded into their fundamental sugar units, can be readily converted into renewable fuels or chemicals [1].

Cellulose degradation is mediated by cellulases, a broad family of enzymes found in all domains of life that hydrolyze (or oxidize) glycosidic bonds between glucose units within cellulose [1, 2]. Natively, multiple types of cellulases work synergistically, but cellobiohydrolases are critical cellulases for breaking down highly crystalline cellulose [3]. Cellobiohydrolases preferentially bind to chain ends and cleave β-1,4-glycosidic bonds between every other glucose unit to produce cellobiose. They are typically processive—remaining bound through multiple cleavage events and moving to follow the shortening chain end [4]. They are specific to either the reducing or nonreducing chain end and cellobiohydrolases of opposite directionality work synergistically to deconstruct cellulose [5].

Cellulases have garnered attention in recent years due to their ability to degrade cellulose-based wastes, such as paper and plant biomass, into fermentable sugars to then produce biofuels. Currently, however, cellulolytic enzymes are too inefficient to accomplish industrial-scale degradation at reasonable cost [6, 7]. In hopes of improving efficiency, scientists have sought to characterize the mechanisms of cellobiohydrolase-mediated degradation to determine structure–function relationships that can be engineered to improve cellulose breakdown rates [8–10].

Cellobiohydrolases are typically composed of one or more carbohydrate-binding modules (CBMs) and at least one catalytic domain (CD). CBMs increase the binding affinity of the enzyme to cellulose and the CD is responsible for glycosidic bond hydrolysis [1, 11]. In some cellulases, these domains are connected by a linker region, which can assist the CBM in substrate binding [12–14].

The mechanism of cellobiohydrolase-mediated processive breakdown of a cellulose chain varies by cellulase family, but the hypothesized multi-step process can be simply outlined as follows: (i) enzyme binding to the crystalline cellulose surface; (ii) decrystallization of a single cellulose chain end from the crystal lattice and insertion into the CD binding tunnel; (iii) hydrolysis to cleave one cellobiose from the chain end; (iv) release of cellobiose into solution, and (v) further chain peeling and sliding through the binding tunnel resulting in forward enzyme motion [4, 10, 15–17]. Processive degradation involves repeated cycling through steps (iii) to (v).

Often, factors that can assist in one step of the degradation cycle will have disparate effects on others. For example, the CBM is instrumental in the binding step (i) but could impede forward motion in the sliding step (v) or, conversely, assist in chain peeling in the initial decrystallization step (ii) and the sliding step (v), depending on the enzyme [18–20]. The cellulose substrate structure is also known to have complex effects at nearly every step [10, 21–23]. Cellulose I, the native allomorph found in plants and algae, can be pretreated with liquid ammonia to form cellulose III. This pretreatment rearranges the hydrogen bond network and lowers the free energy of decrystallizing a cellobiose unit from the crystal lattice, lowering the energetic barrier to chain peeling in steps (ii) and (v) [5, 23]. Cellulose can have varying degrees of crystallinity, and less crystalline substrates have more accessible surface area to enable binding in step (i) [22]. However, both pretreatment and crystallinity affect surface topography and thus the enzyme–substrate binding interface, which can impact multiple steps in unexpected ways [24].

Many studies have aimed to characterize a variety of cellulases from diverse families and design structurally informed mutations in hopes of improving cellulose degradation rates [8, 9, 25–27]. Most studies use bulk biochemical assays, which readily provide ensemble measurement of activity but only measure solution-scale products, necessarily lumping together the functional effects of mutations on specific degradation steps. Single-molecule level investigations of cellobiohydrolases can complement bulk studies and parse out these effects. *Tr*Cel7A, a reducing-end directed cellobiohydrolase from the fungus *Trichoderma reesei*, has been extensively studied using both bulk biochemical assays and single-molecule techniques including atomic force microscopy, optical tweezers, and fluorescence-based single-molecule tracking, each contributing to the overall understanding of the catalytic cycle of *Tr*Cel7A [18, 21, 28–31].

*Tf*Cel6B, a nonreducing-end directed cellobiohydrolase, has garnered interest for industrial-scale use due to its higher thermostability and broader pH optimum compared to other nonreducing-end cellobiohydrolases [9, 17, 26]. *Tf*Cel6B is secreted by the soil bacterium *Thermobifida fusca* and consists of a family 2a CBM and a family 6 CD connected by a flexible linker [3, 17, 26]. The substrate-binding tunnel within the CD is longer and more enclosed than that of other family 6 enzymes and thus is expected to be highly processive, yet single-molecule tracking experiments were previously unable to resolve *Tf*Cel6B’s processive motility [17, 32].

Here, we used optical tweezers to directly observe and measure processive cellulose degradation by *Tf*Cel6B at the single-molecule level. We investigated the impacts of substrate structure on enzyme velocity and bulk hydrolysis rate using *Cladophora* cellulose I_α_ and III (both > 95% crystalline) along with filter paper (~ 60% crystalline) to represent a post-consumer waste feedstock. These three substrates will hereafter be referred to as “CI”, “CIII”, and “FP”, respectively. To investigate roles of the CD and CBM-linker separately, we also produced and assayed “CD only”, a truncated enzyme lacking the CBM and linker, and observed reduced activity compared to that of the whole “intact” enzyme. Additionally, we performed optical tweezers experiments at elevated temperatures and under varied applied loads, yielding information about the energetic barriers of cellulose hydrolysis. Together, this study solidifies *Tf*Cel6B as a processive cellobiohydrolase with strong thermophilic behavior and broadens our understanding of this nonreducing-end directed cellulase.

## Results

### *Tf*Cel6B exhibits bidirectional motility between pauses

**Fig. 1:**
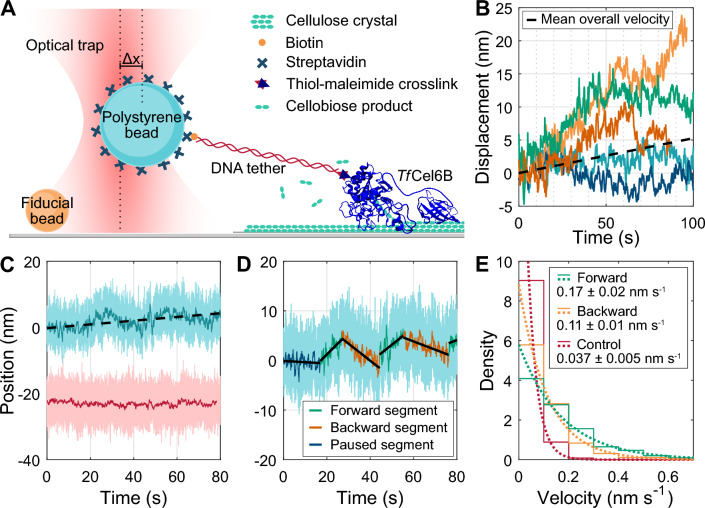
*Tf*Cel6B displays bidirectional motility on cellulose. **A** Representation of optical tweezers *Tf*Cel6B motility assay (not to scale). **B** Sample *Tf*Cel6B motility records on CI at 21 °C demonstrating heterogeneous motility paths. Dashed line represents time-weighted average overall velocity, 0.05 ± 0.03 nm s^−1^ (SEM, N = 22 enzymes). **C** Sample drift-corrected *Tf*Cel6B trace (blue) and the overall velocity fit (0.06 nm s^−1^, dashed black line) compared to a drift-corrected control tether (red, see Additional file 1: Fig. S3). Light colors are raw data (3 kHz), darker overlays are downsampled and averaged. **D** Separation of the record in C into segments of constant velocity. Black lines are linear fits to each segment to determine the segment velocity. Segment colors are defined relative to the overall trace progress direction. Green segments are forward motions, orange backward motions, and blue are considered pauses (absolute velocity < 0.085 nm s^−1^). **E** Step histograms of time-weighted segment velocity probability distributions of intact *Tf*Cel6B on CI at 21 °C from three segmentations, divided into forward (green, *N* = 272) and backward (orange, *N* = 175) populations and overlaid with exponential PDFs (dotted lines), along with 21 °C control (red, *N* = 84) from Additional file 1: Fig. S3. Inset: mean velocities ± 95% confidence intervals from PDFs

Single-enzyme motility studies of *Tf*Cel6B were initiated using an optical trap and tethered bead geometry (Fig. 1A). Polystyrene beads (1.36 μm), conjugated to enzymes via a 1010 bp DNA tether, were introduced to a flow cell containing immobilized cellulose fibers (see “Materials and methods”). A bead was then trapped, calibrated for position and trap stiffness, and positioned above the cellulose substrate. The length of a fiber was scanned below the bead using a piezo stage to facilitate tether formation via *Tf*Cel6B binding. Upon engagement, the stage was held fixed while the bead position was recorded. During the experiment, video was recorded of surface-bound fiducial markers to track and correct for experimental drift in postprocessing data analysis.

When fit with a line, room temperature motility records had overall apparent velocities below 0.1 nm s^−1^ (Fig. 1B, C, Additional file 1: Table S1). However, closer inspection of the records revealed periods of faster forward and backward motility interspersed with apparent pauses, visible in representative traces provided in Fig. 1B and Additional file 1: Fig. S1.

To characterize these phases, motility records were segmented by eye into sections of relatively constant velocity (Fig. 1D and Additional file 1: Fig. S2) and fit to a line using linear least-squares fitting to extract the segment velocity. To avoid bias, each record was segmented three separate times by independent researchers and all resulting segments were combined as one dataset including the three replicates. Segments were sorted into forward moving and backward moving bins defined relative to overall enzyme progress. Segments with durations shorter than 10 s, accounting for less than 15% of the total time observed, were difficult to confidently fit and thus removed from our analyses. Segment velocity distributions, including all three segmentation replicates, were fit to a time-weighted exponential probability distribution function (PDF) using maximum likelihood estimation, yielding means of 0.17 ± 0.02 nm s^−1^ and 0.11 ± 0.01 nm s^−1^ for forward- and backward-moving segments, respectively (Fig. 1E). Mean velocities from each segmentation can be found in Additional File 1: Supp. Table S2.

To conservatively estimate a velocity cutoff for designating segments as paused, we also performed control experiments characterizing instrumental drift. Beads were similarly functionalized with a 1010-bp DNA tether containing a biotin on the free end. Beads were tethered to a streptavidin-coated coverslip surface, trapped, and monitored for position over time (Additional file 1: Fig. S3). Bead position records were drift corrected, segmented in triplicate, and fit in the same manner as cellulase experiments yielding a mean surface-tethered bead drift velocity of 0.037 ± 0.005 nm s^−1^ (Fig. 1E, red). This value is significantly slower than any *Tf*Cel6B dataset (Additional file 1: Fig. S4). Integrating the PDF revealed that 90% of fixed-tether segments would be expected to move less than 0.085 nm s^−1^, and thus we conservatively categorized any segment moving faster than 0.085 nm s^−1^ to be motility and any segment moving more slowly to be a pause.

**Fig. 2:**
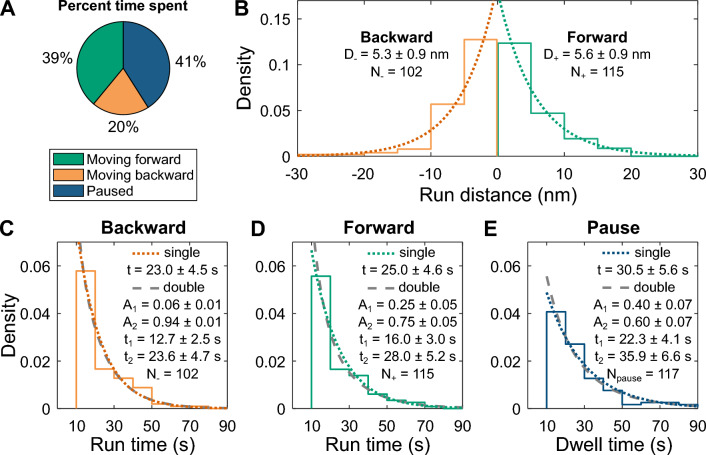
Run and pause distances and dwell times are exponentially distributed. **A** Percent of observation time *Tf*Cel6B was observed in forward, backward or paused states during 3027 s of observation (21 °C, CI, *N* = 22 enzymes). This dataset was segmented in triplicate and utilized for all panels. **B** Run distance probability distributions, split into forward (green) and backward (orange) directions, overlaid with single exponential PDF fits. Insets are means, 95% confidence intervals, and run counts. **C**, **D** Run durations and overlaid single and double exponential PDFs in backward (**C**) and forward (**D**) directions. Insets are fit results ± 95% conf. intervals from two-parameter PDFs, excluding t < 10 s due to segmentation criteria (see Materials and methods). Single exponential PDFs are represented in the same manner as in **B**. Double exponential fits show the amplitudes (A) and mean durations (t) of the two fit populations. **E** Pause durations and fits represented in the same manner as in **C** and **D**

Given this pause criteria, intact *Tf*Cel6B spent 39% of the time in a paused state when assayed on CI at 21 °C. The enzyme spent 41% of the time moving forward (velocity > 0.085 nm s^−1^) and 20% of the time moving backward (< − 0.085 nm s^−1^, Fig. 2A). This dataset includes 22 records spanning 3027 s of observation time. Run lengths and durations were determined by considering periods where the enzyme continuously moved faster than 0.085 nm s^−1^ without reversing direction. The run length probability distributions were exponential (Fig. 2B) with a mean of ~ 5 nm in both directions, equivalent to ~ 5 cellobiose units. Some runs exceeded 15 nm before pausing or reversing direction. We note that, regardless of the direction of force application by the optical trap, the mean run length remained within 5–6 nm (Additional file 1: Fig. S5). Run and pause duration distributions were also fit to single exponential PDFs, yielding means of ~ 20–30 s for all three distributions, all within error of one another (Fig. 2C–E). Run and pause durations additionally were fit to double exponential PDFs using an Expectation–Maximization algorithm, slightly improving the fits.

### Substrate has differing impacts on activity at bulk and single-molecule scales

**Fig. 3:**
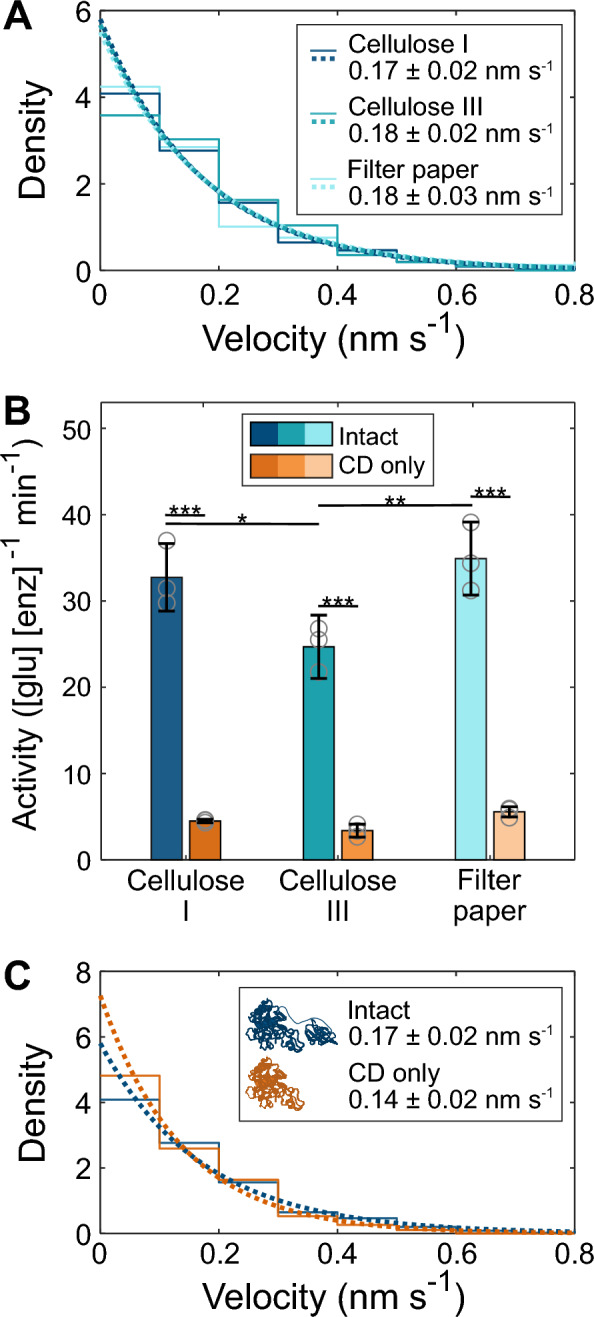
Effects of substrate structure and enzyme domains. **A** Probability distributions and PDFs of intact *Tf*Cel6B forward segments on three substrates. Inset: mean velocities ± 95% conf. intervals (N_CI_ = 272; N_CIII_ = 194; N_FP_ = 106 over three segmentations). **B** Bulk cellobiose production rate by intact or CD only on three substrates. Intact: CI = 32.7 ± 3.9 min^−1^, CIII = 24.7 ± 3.7 min^−1^, FP = 34.9 ± 4.2 min^−1^; CD only: CI = 4.5 ± 0.2 min^−1^, CIII = 3.4 ± 0.8 min^−1^, FP = 5.6 ± 0.6 min^−1^ (error S.D. of 3 replicates). Each replicate is plotted as a gray circle. Lines between datasets indicate means that are significantly different, calculated using Tukey’s Honestly Significant Data test (*α = 0.05; **α = 0.005; *** α = 0.001). **C** Distributions and PDF fits of intact and CD only forward segments on CI. Legend includes representation of domains, mean velocities, and 95% conf. intervals (N_intact_ = 272; N_CD only_ = 106 over three segmentations). Statistical comparisons of datasets shown in all panels can be found in Additional file 1: Fig. S4 and Tables S3 and S4

Based on previous bulk-scale cellobiohydrolase activity assays, we expected to see an appreciable drop in both bulk activity and single-enzyme velocity on cellulose III (CIII) and filter paper cellulose (FP) compared to cellulose I (CI) [22, 33]. Ensemble and single-molecule experiments have both shown, however, that substrate structure affects the speed at which cellulases degrade substrate in unpredictable ways [5, 18, 24]. We assayed three substrate types at both the single-molecule and bulk scale: CI, CIII, and FP (Fig. 3AB, Additional file 1: Fig. S1). Bidirectional motility was seen on all substrates, and single-molecule velocity distributions on each substrate were compared by hypothesis testing via bootstrapping showing no appreciable effects on *Tf*Cel6B velocity in either direction (Additional file 1: Fig. S4). Ensemble hydrolysis assays performed on these same substrates using soluble *E. coli* cell lysates containing heterologously expressed enzymes revealed that CI and FP were degraded similarly over 24 h, but CIII was degraded to a slightly lesser extent (Fig. 3B, statistical analyses in Additional file 1: Tables S3 and S4).

### *Tf*Cel6B isolated catalytic domain is motile, but slower

The relative contribution of the carbohydrate-binding module (CBM) and the catalytic domain (CD) to cellulose catalysis has been shown to vary [18–20]. We hypothesized that the bidirectional motility patterns of *Tf*Cel6B we observed may be due to previously unseen CBM behavior, since the structure of the CD indicates typical unidirectional catalysis [17]. To investigate the contribution of the CBM and linker region to motility and degradation, the isolated CD of *Tf*Cel6B was expressed and purified to produce our “CD only” construct which was then assayed in the same manner as the whole “intact” enzyme. The CD only construct also moved bidirectionally, thus backward motions are not caused by the CBM. While we expected to observe little difference in velocity between the two enzyme forms, as was seen for *Tr*Cel7A using optical tweezers [18], we instead found that CD only moves slower than intact in both directions (Fig. 3C and Additional file 1: Fig. S1). The forward velocity difference is more pronounced when assayed on CIII (39% reduction, *p* < 0.0001), but is still significant on CI (18% reduction, *p* < 0.05) (Additional file 1: Fig. S4). CD only average run distances, run durations, and pause durations were all within error of that of intact (Additional File 1: Fig. S6).

Bulk activity was even more dramatically reduced by removal of the CBM and linker (statistical analysis in Additional file 1: Tables S3, S4). Figure 3B shows the activity of CD only was just 13–16% of intact. It is commonly thought that the CBM is primarily responsible for targeting substrate and allowing initiation of degradation, thus this large reduction in bulk activity is due to difficulty committing to the hydrolysis cycle [11]. However, our complementary single-molecule measurements additionally indicate a slowed hydrolysis cycle after initiation.

### *Tf*Cel6B velocity is minimally impacted by force but unbinds above 10 pN

**Fig. 4:**
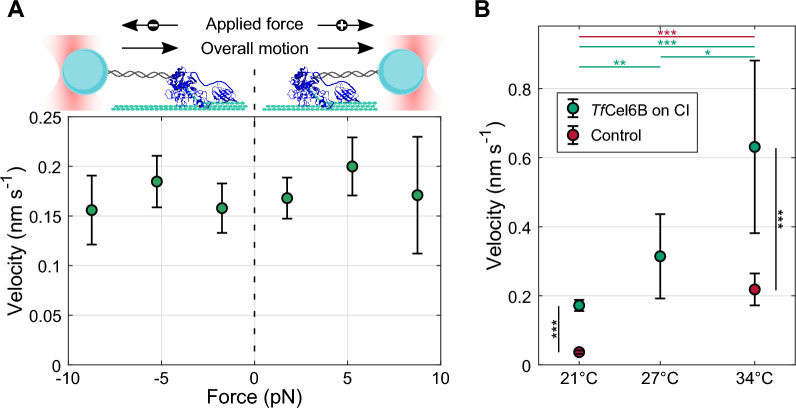
Impact of applied force and temperature on *Tf*Cel6B forward motility. **A** Mean forward velocity of intact *Tf*Cel6B with respect to applied force. To maximize *N*, all three substrate datasets were combined and segmented in triplicate (*N* = 572). Segments were binned by average applied force into 3.5 pN wide bins. Each point is the mean ± 95% confidence interval from the velocity PDF fit within each bin (shown in Additional file 1: Fig. S7). Cartoon depicts the applied force direction relative to forward enzyme motility for opposing (left, negative force) and assisting (right, positive force) loads. **B** Green: average forward velocities of intact *Tf*Cel6B on CI at three temperatures: 21 °C = 0.17 ± 0.02 (N = 272), 27 °C = 0.31 ± 0.12 (*N* = 17), 34 °C = 0.63 ± 0.25 nm s^−1^ (N = 26; error 95% conf. interval). Red: control records after drift correction: 21 °C = 0.037 ± 0.005 (*N* = 84), 34 °C = 0.22 ± 0.05 (*N* = 56). Lines indicate the result of bootstrap hypothesis testing with H_0_: µ_1_ = µ_2_ and H_A_: µ_1_ ≠ µ_2_ (*p < 0.01; **p < 0.0005; ***p < 0.0001). Horizontal lines compare *Tf*Cel6B (green) or control (red) velocity between temperatures and black vertical lines compare *Tf*Cel6B velocity to controls at the same temperature

During all single-molecule motility experiments, the DNA tether was pulled taut applying a range of forces. These forces can be “assisting”, pulling the enzyme in the direction of processive motion, or “opposing”, pulling backward (Fig. 4A, top). All forward segments of intact *Tf*Cel6B at 21 °C on any substrate (CI, CIII, and FP) from all three segmentations were combined and binned based on mean force applied during the segment. A time-weighted PDF was fit to velocities in each bin to calculate the mean velocity under that load (Additional file 1: Fig. S7). Figure 4A shows that forward velocity remains unchanged when subjected to up to 10 pN of either assisting or opposing load. CD only similarly did not show a clear force dependence under loads below 10 pN (Additional file 1: Fig S8). It is of note, however, that attempts to sample intact motility at forces larger than 10 pN were unsuccessful due to *Tf*Cel6B dissociation from the cellulose surface, despite the capability of applying loads over 25 pN in a similar study [18]. A single CD only record persisted under up to 14 pN of assisting load.

### *Tf*Cel6B mean velocity increases with elevated temperature

Bulk cellobiohydrolase activity has been shown to increase with increased temperature, but this could be credited to faster catalysis, easier feeding of chains into the binding tunnel, or reduced time spent bound while not degrading, among other things [26, 32]. We assayed *Tf*Cel6B at 27 °C and 34 °C to determine impacts of elevated temperature on catalysis rate, measured as single-molecule velocity (Fig. 4B, green). Datasets are sparse due to drift challenges in collecting optical tweezers measurements at these increased temperatures. Bootstrap hypothesis testing showed a significant increase in mean velocity between 21 °C and 27 °C (*p* < 0.0005) and a further increase in velocity from 27 °C to 34 °C (*p* < 0.01). To verify this increase was due to increased motility, we performed an enzyme-free control as before at 34 °C (Fig. 4B, red). While unaccounted for drift did increase, it was not nearly as dramatic as the increase in enzyme velocity and mean *Tf*Cel6B velocity remained significantly faster than the control (*p* < 0.0001). One of the many challenges in obtaining elevated temperature measurements was a noticeable decrease in tether lifetime. Many *Tf*Cel6B binding events were briefer than the minimum segment duration of 10 s. The mean bond lifetime of records with durations > 10 s dropped from 157 s at 21 °C to 49 s at 27 °C and only 20 s at 34 °C.

### Single-molecule *Tf*Cel6B motility records include large, fast displacements

**Fig. 5:**
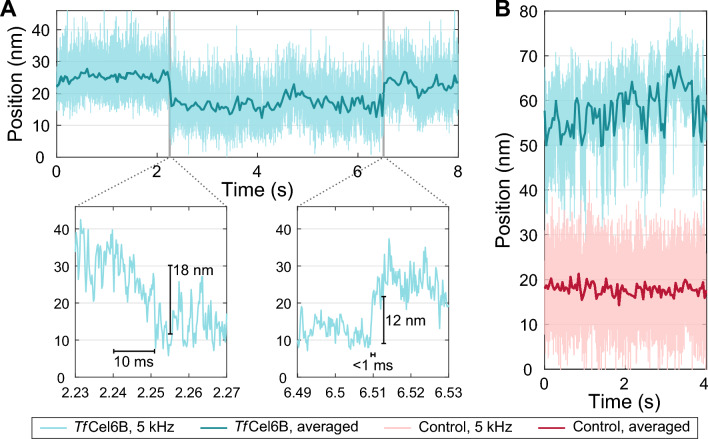
Abrupt extensions and retractions in single-molecule measurements. Examples of fast position changes in motility records. Light coloring is raw data (5 kHz) and dark lines are after averaging and downsampling. **A** Example highlighting two abrupt and distinct position changes. Gray shaded areas are zoomed below showing a slower transition (left) occurring over roughly 50 datapoints spanning 10 ms and a fast transition (right) that occurs within 4 datapoints and 1 ms. **B** Example of a *Tf*Cel6B motility record (top) with multiple large position changes compared to a control record (bottom) with smaller position changes attributed to noise and Brownian motion of the tethered bead in the trap

Throughout many of our motility records, we noticed abrupt positional displacements, such as those shown in Fig. 5 and Additional file 1: Fig. S9. Displacements were observed on all substrates using both intact and CD only constructs but were not present in control traces (Fig. 5B, bottom). Displacements were bidirectional and occurred both with and against the force of the optical trap. Some appeared to reversibly transition between two or more states (Fig. 5B, Additional file 1: Fig. S9). Frequently, displacements were larger than 10 nm and occurred within milliseconds, at times nearing the 2.5-kHz temporal resolution limit of our measurement acquisition settings (Fig. 5A, right).

## Discussion

### Possible models to explain *Tf*Cel6B motility

Motility records of single *Tf*Cel6B molecules were heterogeneous, featuring pauses and segments of slow forward and backward motion on all three substrates assayed using both the intact enzyme and the isolated catalytic domain. The mean forward velocity of 0.17 ± 0.02 nm s^−1^ on cellulose I is similar to the 0.25 ± 0.35 nm s^−1^ velocity of the fungal cellobiohydrolase *Tr*Cel7A measured via optical tweezers in Brady et al. [18]. The velocity of backward *Tf*Cel6B segments was 36% slower on average than that of forward segments across all three substrates. Despite this, the mean distance and duration of backward runs were indistinguishable from forward (Fig. 2). While modest backstepping and pausing of *Tr*Cel7A was observed in Brady et al., bidirectional motility of such an extent observed here for *Tf*Cel6B has not been reported in any other single-molecule study of cellobiohydrolases [18, 19, 21, 28–32, 34, 35].

The conventional model for cellobiohydrolase activity involves localized peeling of a cellulose chain end from a highly organized crystalline lattice and threading the chain end into the enzyme’s catalytic tunnel for hydrolysis (see “Background”). Processive hydrolysis through repetitive bond cleavage along the same cellulose polymer compels a model of unidirectional step-like motion along the chain. While *Tr*Cel7A was observed taking single cellobiose-sized steps consistent with this model, several observations here suggest there is more complexity underlying cellulose degradation by *Tf*Cel6B. Our measurements provide constraints to possible models of *Tf*Cel6B motility including: (a) similar magnitude of forward and backward velocities implying similar mechanisms underlying both motions (Fig. 1E); (b) run lengths averaging ~ 5 nm and at times exceeding 10 nm (or multiple catalytic cycles) in both directions regardless of force application direction (Fig. 2B, Additional file 1: Fig. S5); (c) minimal velocity sensitivity to substrate ultrastructure (Fig. 3A); (d) insensitivity of velocity to the magnitude and direction of applied force (Fig. 4A); and (e) presence of rapid transitions on the order of 5–20 nm, equivalent to 5–20 cellobiose units (Fig. 5).

**Fig. 6:**
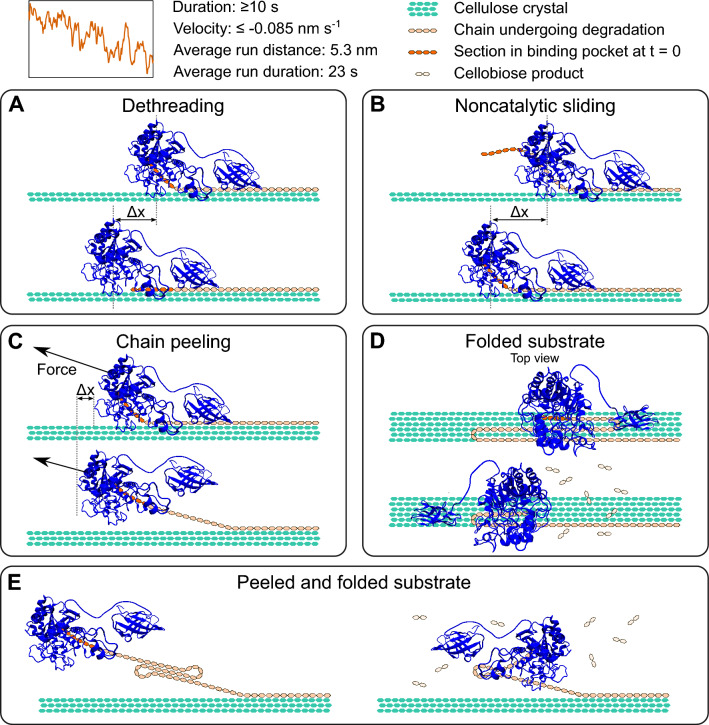
Potential mechanisms for slow backward motions. Note in these cartoons overall progress is towards the right and backward motion is to the left. Backward motion is denoted by Δx. Top: trace showing an example of a slow backward motion and list of characteristics of these motions. **A** The cellulose chain dethreads from the substrate-binding tunnel. **B** The cellulose chain has slipped through the back of the substrate-binding tunnel and the enzyme must slide backward to reengage the nonreducing chain end. **C** Assisted by the optical trap, the chain is peeled away from the cellulose surface resulting in apparent backward motion. **D** Bends in a reorganized substrate-associated cellulose chain require the enzyme to change direction of motion while continuing to hydrolyze from the nonreducing end. **E** A peeled chain folds upon itself, and unidirectional hydrolysis along the chain appears as back-and-forth motion

We considered multiple mechanisms that might manifest as apparent backward motility, summarized in Fig. 6. One possibility to produce modest backward motion is through dethreading of a bound chain from the substrate-binding tunnel (Fig. 6A). This tunnel, however, can only accommodate three cellobiose units, or roughly 3 nm of cellulose chain contour, at a time which is inconsistent with observation (b) [17].

Another possibility is noncatalytic sliding of the chain forward through the tunnel exit (Fig. 6B). *Tf*Cel6B has two flexible loops covering the binding tunnel exit, but molecular dynamics simulations have shown the loops readily open when *Tf*Cel6B is adsorbed to a crystalline cellulose surface [10, 17]. Chain slipping through the open loops followed by backsliding to reengage the chain end and restart processive degradation would lead to records consistent with backward motions. Without catalysis, however, no energy source is available for the ‘slip’ or ‘reengage’ phase to perform work against the optical trap, which is not consistent with the force symmetry in observation (b). Slippage through the tunnel would also enable hydrolysis along random points in the chain producing cellodextrin products much larger than cellobiose, which have not been observed [9].

Apparent backward motion could also be caused by a cellulose chain slowly peeling from the crystal lattice, depicted in Fig. 6C. Observation (b), however, argues against peeling, as geometric considerations would cause disparate run lengths depending on load orientation. Given our assay design, 10 nm of chain peeling would cause ~ 3 nm or ~ 23 nm of apparent backward motion in classical opposing and assisting load orientations, respectively (Additional file 1: Fig. S10). Peeling is also expected to be dependent on force and substrate structure [23], which is inconsistent with (c) and (d). While our results suggest that slow peeling of the chain is not the cause of apparent backward motility, rapid chain peeling could underlie the large jumps of observation (e), which is discussed below.

An additional substrate-mediated explanation for slow backward motions is irregularity of the cellulose chain on the surface of the crystal lattice (Fig. 6D). While cellulose crystals consist of chains tightly packed in parallel bundles, single chains outside of a crystal lattice were shown to fold and self-associate, creating switchbacks in the cellulose track [36]. Surface-exposed chains may reorganize into switchbacks during substrate purification steps using acid/base treatment or mechanical processing, then reanneal to the crystal surface. Self-associated chains could also be peeled from the crystal surface (Fig. 6E). Apparent backward motions would be observed when *Tf*Cel6B degrades sections of chain oriented anti-parallel to the crystal lattice, but processive hydrolysis would underlie both directions of motility, in agreement with (a). In this model, the modestly dampened velocity of backward motility may be explained by chain organization altering the enzyme–substrate interface [10].

**Fig. 7:**
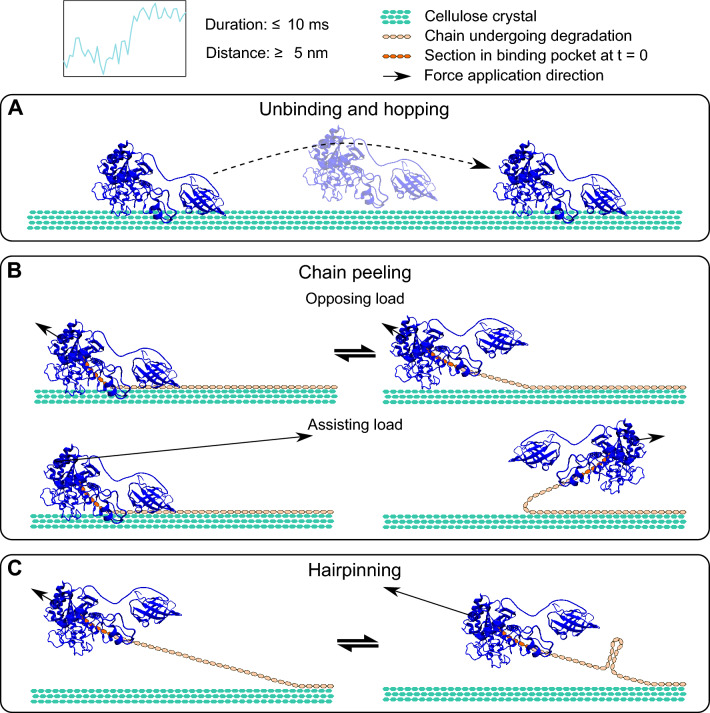
Potential mechanisms for fast position changes. Top: example of a fast position change and listed characteristics of these motions. **A** *Tf*Cel6B unbinds from the cellulose surface, diffuses in solution, and rebinds elsewhere. **B** The chain is peeled from the cellulose surface, similarly to Fig. 6C, but at a much faster rate. In the opposing load orientation, hydrolysis after peeling progresses forward towards the right as drawn, but in the assisting load orientation, hydrolysis appears as backward motion. **C** A peeled cellulose chain self-associates, creating reversible hairpin folds in the chain that abruptly reduce the tether contour length

The large, rapid position changes noted in observation (e) require considering mechanisms much faster than catalytic cellulose degradation. The ubiquitous abrupt displacements occur within milliseconds and can represent 5 or more catalytic events making them too fast to be attributed to enzymatic activity [32]. In some cases, displacements were reversible; examples in Additional file 1: Fig. S9 show the enzyme appearing to toggle between two or more positions. We detail three possible explanations: diffusion-based unbinding and hopping, chain peeling from the substrate, and hairpinning (Fig. 7).

Diffusion-driven unbinding and hopping of this size is consistent with the observed timescales and magnitudes (Fig. 7A) [32]. However, it could not occur against the load of the optical trap and thus is inconsistent with the bidirectional symmetry observed. This mechanism is also not consistent with traces that show reversible toggling, as reversibility implies the enzyme remains associated with the substrate.

In addition to *Tf*Cel6B moving along the surface, position changes may also occur from changes in the tether’s contour length such as from rapid peeling of a single enzyme-bound cellulose chain off the crystalline surface (Fig. 7B). A peeled chain remains in proximity to the rest of the cellulose fibril, and thus reversible re-association with the fibril, particularly in the opposing load orientation, is consistent with our observations (Fig. 7B, top). In the assisting load orientation (Fig. 7B, bottom), returning to the original strand orientation is unlikely, but chain folding and re-association to the surface would cause a switchback as in Fig. 6D to help explain backward movement. Degradation of a chain peeled in the opposing load direction would appear as forward motility, and in the assisting load direction would appear backward.

Folding of a peeled chain would also manifest as abrupt contour length changes. As shown in Fig. 7C, the peeled chain could reversibly self-associate to form hairpin-like microstructure and shorten the contour length. This mechanism has been observed for single cellulose chains under similar loads [36]. Likely, a combination of substrate peeling and hairpinning mechanisms, seen as fast transitions, reorganize the chain, further motivating our substrate-driven models of slow backward motility (Fig. 6D–E).

Despite nearly identical assay conditions, Brady et al. did not report these fast displacements when assaying *Tr*Cel7A, but we theorized that they may have been present and simply overlooked [18]. Upon reexamination of motility records collected in that study, we indeed found examples of displacements > 5 nm over very short timescales, although large displacements appeared to occur less frequently for *Tr*Cel7A than for *Tf*Cel6B (Additional file 1: Fig. S11). Substrate conformation could therefore be driving the motions for both enzymes, but the distinct cellobiohydrolases likely interact with their substrate differently thus displacements are seen to different extents. If large displacements are indicators of substrate reorganization enabling bidirectional motility, it is unsurprising that *Tr*Cel7A motility is largely unidirectional [18, 29]. Further investigations into the *Tf*Cel6B–cellulose interface are required to test this hypothesis.

### Single-molecule measurements decouple binding step from hydrolysis cycle

Ensemble-scale biochemical assays are fast and effective at measuring the total soluble sugar (i.e., cellobiose) production rate of cellulases under a variety of assay conditions but muddle together binding, hydrolysis and release steps. Our single-molecule assay, however, tracks enzyme progress along the substrate, decoupling the steps and enabling direct measurement of the impact of varied conditions on hydrolysis rate. We used these two approaches together to glean valuable insights into the role of the CBM and impacts of substrate structure on *Tf*Cel6B activity.

Previously, we reported a 3.5 × reduction in bulk activity of *Tf*Cel6B on Avicel cellulose III compared to cellulose I, despite the expectation that cellulose III would be more easily degraded due to the lower energy barrier to peeling a chain off the crystal lattice [23, 33]. We observed here a more modest, albeit noticeable, reduction in bulk activity from the conversion of *Cladophora* cellulose I (CI) to cellulose III (CIII) (Fig. 3B, p < 0.05). This trend was not reflected in single-enzyme velocity, however, with CI and CIII having nearly identical velocities (Fig. 3A), indicating intact *Tf*Cel6B is similarly efficient at processively hydrolyzing CIII after initiating degradation and the reduction in bulk activity is likely due to difficulties in this first step [24, 37].

We investigated the link between crystallinity and enzyme activity for *Tf*Cel6B using filter paper (FP) as a substrate, which is roughly 25% less crystalline than CI and CIII and represents recycled paper as a feedstock. Both bulk activity and single-enzyme velocity were unchanged between FP and CI (Fig. 3AB, Additional file 1: Fig. S4). This was unexpected, as reduced crystallinity is thought to increase the fraction of substrate-bound enzymes, however, disruptions to the crystal structure could subtly impact the enzyme–substrate interactions that help to facilitate degradation initiation [10, 22]. These competing factors are an example of intricacies of enzymatic hydrolysis that can be elucidated using multiscale approaches including single-molecule methods. Quantification of binding and dissociation rate constants in future studies can determine if substrate impacts these factors.

Differing hypotheses exist as to the role of the carbohydrate-binding module (CBM) and linker in the cellulose hydrolysis cycle. While many conclude that the CBM’s primary purpose is increasing the affinity of enzymes for cellulose, it is debated as to whether the CBM and linker participate in the hydrolysis cycle [3, 18–20, 28]. We demonstrate that removal of the family 2a CBM and linker region of *Tf*Cel6B decreases enzyme velocity by up to 39% (Fig. 3C). Without this context, significantly reduced bulk activity of CD only (Fig. 3B) would be blamed on reduced ability to initiate degradation, but the velocity decrease indicates the CBM and linker also assist in other steps of the processive catalytic cycle.

### Contextualizing *Tf*Cel6B mechanism to other processive cellulases

Prior to this study, processive degradation of cellulose by *Tf*Cel6B has not been directly observed at the single-molecule level. We sought, therefore, to compare our results to studies of similar enzymes. The most well-studied cellobiohydrolase on the single-molecule scale is the fungal *Tr*Cel7A and thus is compared here most frequently.

The contribution of *Tf*Cel6B’s CBM2a and linker to hydrolysis starkly contrasts with *Tr*Cel7A atomic force microscopy and optical tweezers studies in which removal of the family 1 CBM and linker did not inhibit motility [18, 28]. Looking within family 6 cellobiohydrolases, however, *Tr*Cel6A (family 1 CBM) and *Cf*Cel6B (family 2a CBM), have been shown to lack long-lived processive activity when lacking CBMs [19, 35]. Additionally, the family 2a CBM from a *Cellulomonas fimi* endocellulase has been shown to disrupt the crystalline cellulose surface and is hypothesized to assist the CD by ‘lifting’ cellulose chains to aid hydrolysis [20]. For one cellobiohydrolase hydrolysis cycle (described in detail in Background), it is usually assumed that a single cellobiose unit is decrystallized from the surface in step (v). Destabilization of a larger section of cellulose chain by CBM2a could provide a shortcut through the cycle for multiple sequential catalytic events. Altogether, previous studies and observations presented here indicate that the role of the CBM varies greatly depending on the enzyme structure and family.

Similarly to *Tr*Cel7A, the mean forward velocity of *Tf*Cel6B was minimally impacted by force over a range from -10 pN to 10 pN, suggesting that the motility cycle is biochemically dominated and not mechanically limited (Fig. 4A) [18]. Despite this, intact *Tf*Cel6B appears to grip weakly, dissociating under loads exceeding 10 pN. This result was unexpected, given that *Tr*Cel7A has a similarly long binding pocket and was observed processing against more than 20 pN of opposing load using a nearly identical assay and instrument as in the present study [17, 18]. However, other family 6 cellobiohydrolases are also known to bind more weakly to cellulose than *Tr*Cel7A [10, 34, 35]. Weak binding may be an evolutionarily advantageous trait: in enzyme mixtures with multiple cellobiohydrolases degrading in opposing directions, head-on collisions of enzymes are expected and family 6 enzymes may have evolved to more readily dissociate to “yield” to opposing enzymes and avoid long unproductive stalls. This behavior was observed by Uchiyama et al. when *Cf*Cel6B was seen dissociating upon head-on collisions with *Tr*Cel7A [34].

*Tf*Cel6B is of interest for industrial use due to its thermostability, remaining fully active up to 55 °C [26]. Preliminary measurements comparing enzyme motility at 21 °C, 27 °C, and 34 °C indicate a significant velocity increase with each temperature increase (Fig. 4B). *Tf*Cel6B tended to dissociate more quickly from the substrate at elevated temperatures, consistent with single-molecule tracking measurements that observed a 20% decrease in tightly bound *Tf*Cel6B molecules when temperature increased from 23 °C to 45 °C [32]. Another single-molecule tracking study of *Tr*Cel7A suggested that chain dethreading and dissociation can only occur in the “poised” state, in which a glycosidic bond has been cleaved and product released but substrate has not yet threaded through for the next catalytic step. At high temperatures, the loops at the tunnel exit of *Tf*Cel6B would be more flexible and facilitate faster product release, which could bias the poised state and increase the dissociation rate if a similar dissociation mechanism exists for *Tf*Cel6B [38]. While surface-coupled optical tweezers measurements at elevated temperatures present challenges associated with instrumentation stability and drift, measuring cellobiohydrolase-mediated degradation under more native temperature conditions could help us further understand the mechanism of thermophilic cellulases.

## Conclusions

Cel6B from *Thermobifida fusca* has the potential for implementation in industrial-scale cellulosic biofuel production, but direct observation of processive motility by *Tf*Cel6B has been unsuccessful prior to this study. Here, we track single *Tf*Cel6B molecules degrading cellulose using optical tweezers. Unlike the expected unidirectional motility, records contained both forward and backward motion and frequent pauses interspersed with abrupt bidirectional position changes. We propose several explanations for these noncanonical records and hypothesize that substrate irregularities, possibly brought about by *Tf*Cel6B unconventionally interacting with cellulose, underlie these observations.

Some of our hypotheses, such as chain peeling, are assisted by the optical trap and not expected to occur to this extent natively. Nevertheless, the ability of *Tf*Cel6B to degrade cellulose with apparent abnormal substrate geometries indicates that it is highly processive, albeit slow. *Tf*Cel6B seems to be an ideal “teammate” for enzyme mixtures. In addition to adapting to diverse substrate terrain, it yields and dissociates from substrate at the relatively low force of ~ 10 pN which may be beneficial when faced with collisions with other enzymes.

Our measurements of purified *Tf*Cel6B lay the groundwork for future efforts to engineer improved enzymes for use in biofuel production. Our observations of shortened bound lifetime at elevated temperatures indicate that increasing processivity may be a means to improve ensemble degradation rates. Our finding that the CBM contributes to the processive hydrolysis cycle suggests mutative studies of CBM2a may be fruitful, especially when paired with single-molecule measurements of top-performing *Tf*Cel6B-CBM2a mutants. Furthermore, while we measured changes in degradation velocity after initiation of degradation, complementary assays investigating binding and dissociation behavior under conditions tested here would yield a more complete view of the hydrolysis mechanism. While the studies here constitute a minimal system isolating a single enzyme or even sub-components of an enzyme, similar studies in the presence of synergistic enzymes will help elucidate how a cocktail of cellulose-degrading enzymes work as a system. Altogether, our results characterize enzyme motility and hydrolysis on multiple relevant substrates and solidify *Thermobifida fusca* Cel6B as a robust and processive enzyme with broad opportunities for mutational studies and immense promise for implementation in cellulosic biofuel production processes.

## Materials and methods

### Cellulose purification and preparation

Cellulose I_α_ was purified from *Cladophora sp.* (*Cladophora glomerata*) using previously published methods [18]. Cellulose III was created from cellulose I by anhydrous liquid ammonia pretreatment, as described before [39]. Filter paper substrate was prepared by cutting Whatman grade 1 filter paper into 1 mm^2^ pieces. For optical tweezers assays, all substrate samples were wet and mechanically broken up using a tissue homogenizer to enable loading into flow channels. For bulk hydrolysis assays, cellulose I and III were used as-is following purification, and filter paper was frozen using liquid nitrogen, milled using a coffee grinder, then dried at 80 °C for 30 min prior to enzymatic digestion.

### Enzyme expression and purification

All proteins in this study were encoded in pET26b(+) vectors and expressed in BL21 competent cells using autoinduction [40]. Glycerol stocks for both the intact full-length enzyme and isolated CD were used to inoculate 10 mL of LB media with 50 µg mL^−1^ kanamycin and grown overnight at 37 °C with 200 rpm orbital shaking. After 16 h, seed cultures are expected to have reached their exponential growth phase (0.5–0.6 OD600) and were transferred to 250 mL of Studier’s autoinduction medium (TB + G) with 50 µg mL^−1^ kanamycin [40]. Cultures were incubated at 37 °C for 6 h to return once again to an exponential phase before autoinducing at 20 °C for 20 h then 16 °C for 20 h. Cells were then pelleted via centrifugation at 30,000 × *g* for 10 min at 4 °C and lysed according to their application.

For bulk assays and ELISA, 0.5 g of cells were isolated from the dry cell pellet and mixed with 2.5 mL lysis buffer I or II, 35 µL protease inhibitor cocktail (1 µM E-64, Sigma Aldrich), and 2.5 µL lysozyme (Sigma Aldrich). Lysis buffer I (20 mM NaOAc, 10 mM NaCl, 20% glycerol, pH 5.5) was used for bulk degradation assays, but lysis buffer II (20 mM Na_3_PO_4_, 10 mM NaCl, pH 7.4) was used for ELISA assays due to sensitivity to pH and glycerol. Cells were then sonicated using a Qsonica Q700 with a 1/8″ microtip for 1 min (20% amplitude, 5 s pulse intervals, 30 s pauses). Finally, the sample was centrifuged at 15,500×*g* for 45 min to pellet the insoluble cell debris and the supernatant containing soluble cell lysate was used in bulk activity assays or ELISA.

For single-molecule assays, lysis steps were altered and further purification steps were required. For every 3 g of dry cell pellet, 15 mL lysis buffer III (20 mM Na_3_PO_4_, 500 mM NaCl, 20% glycerol, pH 7.4), 200 µL of protease inhibitor cocktail, and 15 µL of lysozyme were added. The lysis mix was vortexed to resuspend before sonication using a 1/4″ microtip for 2.5 min (20% amplitude, 10 s pulse intervals, 30 s pauses) on ice. Insoluble cell debris was pelleted via centrifugation at 15,000×*g* for 1 h and the soluble lysate supernatant was syringe filtered (0.45 µm pore size) prior to purification. *Tf*Cel6B was isolated from the crude soluble lysate by immobilized metal affinity chromatography (IMAC) using a Histrap FF Ni^2+^–NTA column (Cytiva) equipped to a BioRad NGC FPLC. The column was first equilibrated with roughly 5 column volumes (25 mL) buffer A (100 mM MOPS, 500 mM NaCl, 10 mM imidazole, pH 7.4) then the entire volume of lysate was loaded at a rate of 1 mL min^−1^. The column was then washed with 25 mL buffer A until a stable baseline was achieved via in-line absorbance measured at 280 nm. *Tf*Cel6B was then eluted using buffer B (100 mM MOPS, 500 mM NaCl, 500 mM imidazole, pH 7.4). Enzyme yielded from this first step of purification is only ~ 70% pure on average, thus a second purification step is required. Anion exchange (AEX) was performed using a HiTrap Q HP Sepharose AEX column (Cytiva) equipped to a BioRad NGC FPLC. The AEX column was first washed with 3 column volumes (15 mL) 2 M NaCl, then 15 mL 2 M NaOH, and finally 500 mL DI water. The column was then equilibrated with 25 mL AEX start buffer (20 mM Tris, pH 8), before loading IMAC eluents from the first purification step that were dialyzed into AEX start buffer. The column was then washed with 25 mL AEX start buffer at 1 mL min^−1^ and finally eluted by flowing through 150 mL of start buffer at 1 mL min^−1^ with a gradient of 0–25% AEX elution buffer (20 mM Tris, 2 M NaCl, pH 8). Fractions were continuously collected over the entire elution period in 3-mL portions corresponding to peaks in the in-line A280 absorbance. Fractions that contain highly pure *Tf*Cel6B were identified using SDS-PAGE and dialyzed into 10 mM NaOAc, pH 5.5 buffer for use in single-molecule assays.

### Bulk activity assays

Soluble cell lysate was used for bulk activity assays due to low yields after protein purification steps. The amount of recombinant His-tagged enzyme in each lysate was measured using a His tag ELISA detection kit (GenScript) following manufacturer’s protocols. These ELISA kits have a detection range of 729 ng of His-tagged protein per 1 mL sample, thus cell lysates were diluted tenfold in lysis buffer II. His-tagged protein concentration was estimated by comparing raw absorbance values to BSA standards.

For each enzyme/substrate combination, 10 mg of substrate was wetted with 0.5 mL acetate buffer (50 mM, pH 5.5) before adding 0.5 mL crude cell lysate of cells expressing either intact *Tf*Cel6B or the isolated catalytic domain. The samples were then incubated at 60 °C accompanied by 1000 rpm orbital shaking. After 24 h, the amount of glucose equivalents released into solution was estimated by dinitrosalicylic acid (DNS) assay and compared to glucose standards [41]. Activity was then normalized based on enzyme content in each reaction mixture.

### Optical tweezers assay preparation

Beads were functionalized with cellulase as previously described [18]. First, intact or CD only enzyme constructs were maleimide-activated using sulfo-SMCC (ThermoFisher Scientific) according to manufacturer instructions. Excess sulfo-SMCC was removed using a 30 kDa cutoff chromatography column (Bio-Rad) before adding biotin-1010 bp-thiol DNA in a 1:10 molar ratio of DNA to enzyme and incubating for 30 min. Streptavidin-coated polystyrene beads (1.36 µm, Spherotech, Inc.) were then added such that there are 1.5 × more beads than DNA molecules. Bead concentration was calculated based on % w/v, density, and mean diameter. DNA concentration was measured with a UV–Vis spectrophotometer. After the solution was rotated end over end at roughly 40 rpm for 45 min at room temperature, biotin-labeled bovine serum albumin (BSA, Sigma-Aldrich) was added to block unoccupied binding sites on the beads and the solution was rotated for another 20 min. Finally, the beads were washed and resuspended in 50 mM acetate buffer, pH 5.5.

Optical tweezers experiments were prepared as described previously [18]. A flow channel was filled with the substrate of interest and dried at 90 °C for 30 min to adhere substrate to the coverslip. After the slide cooled, 0.005% (w/v) 0.7 μm polystyrene beads (Spherotech, Inc.) in 50 mM acetate buffer were introduced to the flow channel and allowed to adhere for 5 min creating fiducial markers. Then, 5 mg mL^−1^ BSA (EMD Millipore Corp.) in acetate buffer was introduced and left for 15 min to block nonspecific binding. Finally, functionalized beads were introduced and the channel was sealed for imaging.

### Optical tweezers data collection

To measure single-enzyme motility, a functionalized bead was trapped with a 1064 nm laser and calibrated for position and trap stiffness. The bead was suspended above a cellulose filament and the stage moved to scan the filament beneath the bead until the enzyme bound, pulling the DNA tether taut and displacing the bead from the trap center. The position of the bead was then collected at either 3 or 5 kHz with simultaneous lowpass filtering at or below the Nyquist frequency until the bond dissociated causing the bead to fall back into the trap center. During data collection, video of the sample plane was recorded at 0.5 to 1 Hz.

All experiments were carried out at 21 °C, other than the elevated temperature experiments at either 27 °C or 34 °C which were achieved using localized heating at the slide as well as increasing the temperature of the insulated chamber surrounding the microscope. The chamber, constructed of acrylic, was brought to temperature using an Air-Therm heater (World Precision Instruments, Inc.) prior to experimentation and between periods of data collection. The AirTherm was switched off during calibration and data collection to reduce noise. To minimize heat loss, the chamber was lined with dry heating pads (CVS Pharmacy, Inc). To provide localized heating, the top of the slide was covered with a 90 mm × 40 mm × 3 mm aluminum plate containing a 30 mm hole in the middle for condenser lens access and flexible heating pads (2.5 W, 1″ × 3″, Benchmark Thermal) were affixed to the aluminum plate. Two thermocouples (Omega) were imbedded in the aluminum plate: one to display the temperature during experiments and one connected to a PID controller (Micromega, CN77000 Series) to modulate current to the flexible heating pads.

### Motility data analysis

Bead position records were first decimated to 2.5–3 kHz, if not already, before averaging with an exponentially weighted time moving average with a window size of 20 points. The record was then downsampled by a factor of 100 before drift correction and segmentation.

As published previously, a region of interest (ROI) surrounding one fiducial marker from the sample plane video was analyzed using a cross-correlation-based position tracking algorithm [18, 42]. To improve accuracy, an ROI of a second marker was analyzed and the trajectories averaged together. The combined drift record was then smoothed and subtracted from the bead position record. Because this method is not well-suited to account for drift perpendicular to the sample plane, records that contained Z-drift to a degree noticeable by eye were removed from the dataset.

Due to the nonlinearity of motility records, all records were segmented into sections of constant velocity by eye. To avoid bias, each record was segmented three times by independent researchers blinded to the experimental conditions of the record. All segmentations were utilized such that each record appears in the dataset three times, segmented in three ways. A line was fit to each segment in MATLAB using linear least-squares fitting and the slope was taken as the mean velocity over the segment. The distance traveled during a segment was calculated by multiplying the velocity with the duration of the segment. The sum of distances traveled across all segments within a record was used to determine the direction of overall enzyme progress; segments that progressed in the same direction were deemed “forward” and positive velocity, and segments that progressed opposite to overall trace progress were “backward” and negative velocity.

Exponential probability distributions were fit using MATLAB’s Statistics and Machine Learning Toolbox, which utilizes maximum likelihood estimation (MLE), over the range of ${0}\le{\text{x}}<{\infty}$, outputting the mean and 95% confidence interval of the distribution. This method does not require binning, but bins are used to visually represent the distributions for the reader. The density within each bin is calculated as the fraction of observations within the bin divided by the bin width. Segment velocity distributions were time-weighted by repeating the velocity for N_R_ observations, with N_R_ equal to the segment duration divided by 10 s rounded down to the nearest integer. We selected 10 s as the weight basis for two reasons: 1. shorter durations artificially shrank fit confidence intervals; and 2. longer durations exceed the minimum segment time. Reported N values in the text do not include replicated observations due to weighting, but do include segmentation replicates. Mean velocities from each segmentation replicate can be found in Additional file 1: Table S2.

Due to the minimum segment duration, run and pause duration probability distributions were fit with a two-parameter exponential distribution in which the total probability in the domain ${10}\le{\text{t}}<{\infty}$ is equal to 1. Double exponential PDFs were fit to the data using a custom Expectation–Maximization algorithm with a tolerance of 1E−5 s.

### Enzyme-free controls

Control experiments were designed to measure drift present after drift correction and determine the minimum velocity that can confidently be labeled as enzymatic motility. Fiducial beads, with the addition of 0.04 mg mL^−1^ streptavidin (EMD Millipore Corp.) in solution, were adhered to the surface as before. Blocking buffer (5 mg mL^−1^ BSA, 3 mg mL^−1^ casein) was allowed to adhere for 20 min before biotin-1010 bp-digoxigenin DNA was added and allowed to bind for 20 min. Finally, 1.23 μm carboxylated polystyrene beads (Spherotech) coated with anti-digoxigenin (Roche Diagnostics) using EDC chemistry (Thermo Scientific) were flowed into the channel. To collect control records, a bead was centered in the detection zone, trapped, and the stage moved to pull the DNA tether taut. Bead position and sample plane video was collected as described above. Upon tether rupture, the bead was calibrated for position and force. This same procedure was repeated at 34 °C using the temperature control methods described above.

## Supplementary Information

Additional file 1.

## Data Availability

All data generated during this study are included in this article and its supplementary information files. Raw data are available from the corresponding author upon reasonable request.

## Abbreviations

CBM: Carbohydrate-binding module
CD: Catalytic domain
CI: *Cladophora* Cellulose I_α_
CIII: *Cladophora* Cellulose III
FP: Filter paper cellulose
CD only: Isolated catalytic domain of *Tf*Cel6B lacking the carbohydrate-binding module and linker region
Intact: *Tf*Cel6B with all associated domains (catalytic domain, linker, and carbohydrate-binding module)
PDF: Probability distribution function
