# Supplementary material for: Thermobifida fusca Cel6B moves bidirectionally while processively degrading cellulose
Source: Biotechnol Biofuels Bioprod. 2024 Dec 4;17:140. doi: 10.1186/s13068-024-02588-0 (PMC11616356; doi:10.1186/s13068-024-02588-0)
Supplement: Supplementary file 1 — Additional file 1. [file 13068_2024_2588_MOESM1_ESM.docx]

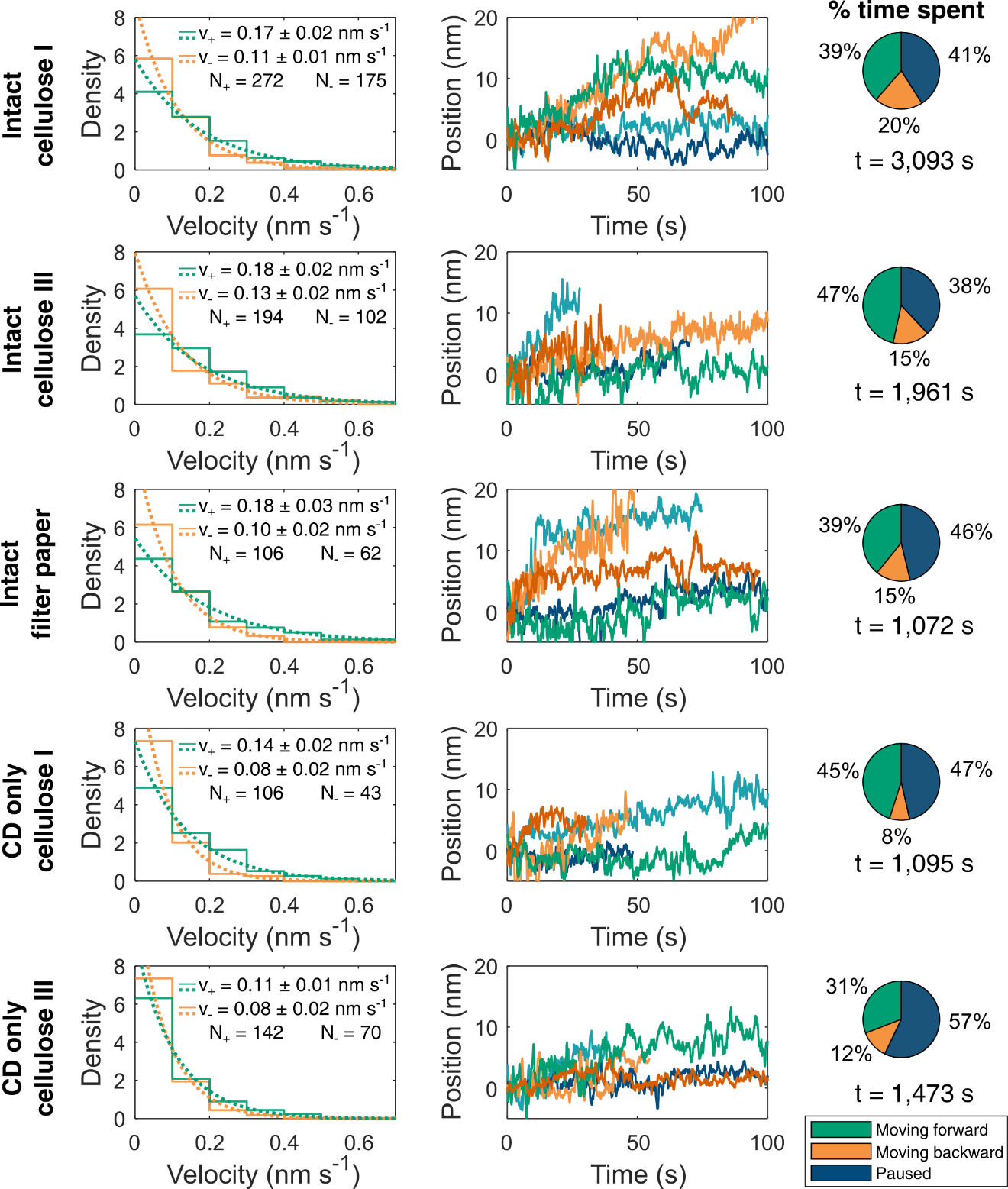


**Fig. S1. Motility distributions of single-molecule *Tf*Cel6B datasets.** From left to right: Enzyme form and substrate; segment velocity probability density step histograms (solid lines) and overlaid exponential probability density functions (dashed lines) separated into forward (green) and backward (orange) segments with inset means and 95% confidence intervals from PDFs, with each trace segmented in triplicate; representative examples of drift-corrected motility records; pie charts of percentage of time spent in each state of motility. Time totals (t) are the observation time after removing segments under 10 s averaged across three segmentations.


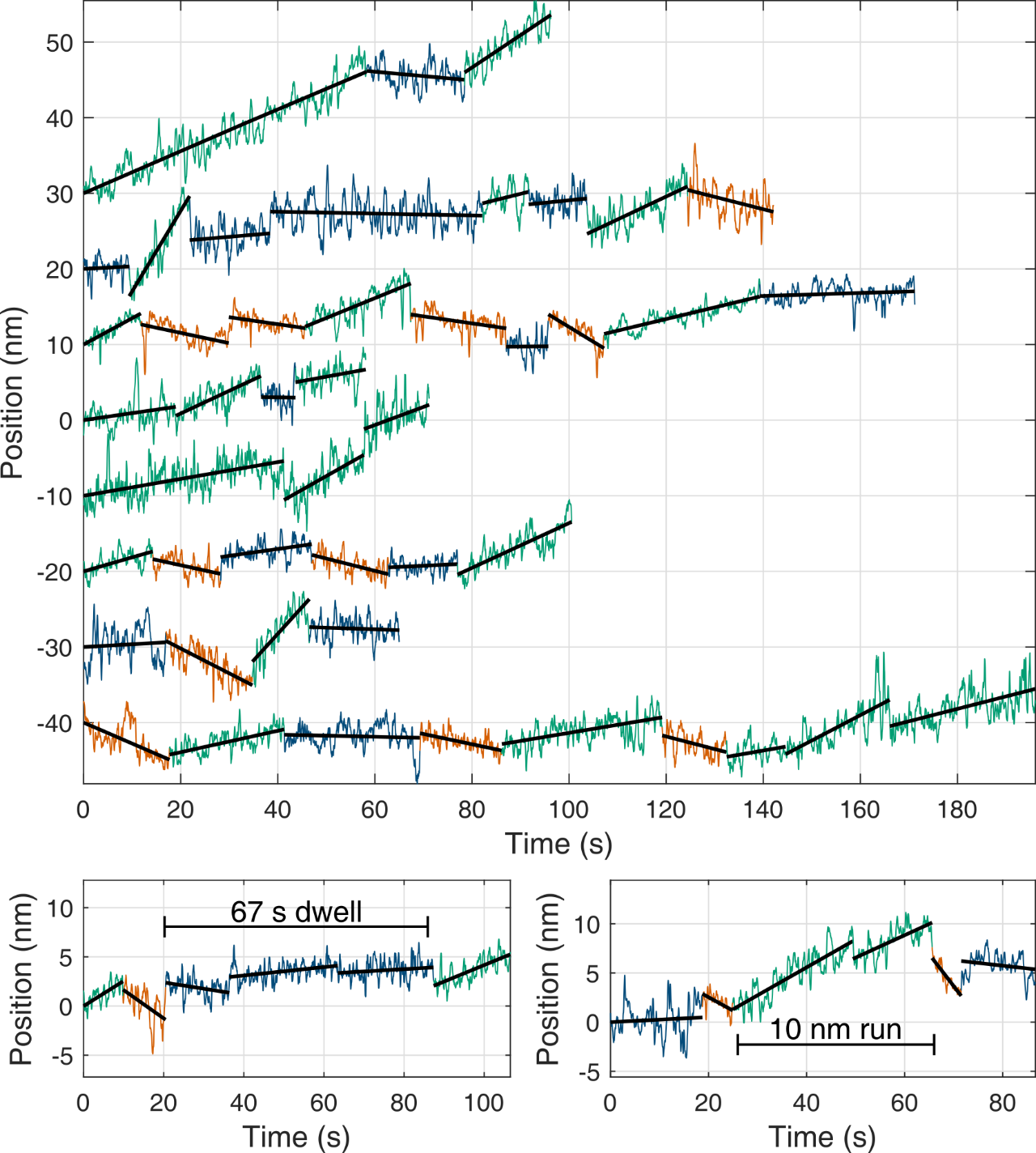


**Fig. S2. Segmentation examples.** Top: Representative *Tf*Cel6B motility records and segmentation, offset for clarity. Records were divided by eye into sections of constant velocity, then segments were fit with a line (black overlay) to determine average velocity. Segments in blue are moving at a speed slower than 0.085 nm s^-1^ and considered paused, segments in green are moving forward, and in orange are moving backward. Distance travelled during a segment is calculated by multiplying duration and velocity. Bottom, left: Example of a pause dwell composed of multiple segments. Bottom, right: Example of a run, with multiple forward moving segments in sequence. The distance travelled during each segment is summed to determine the run distance.

**
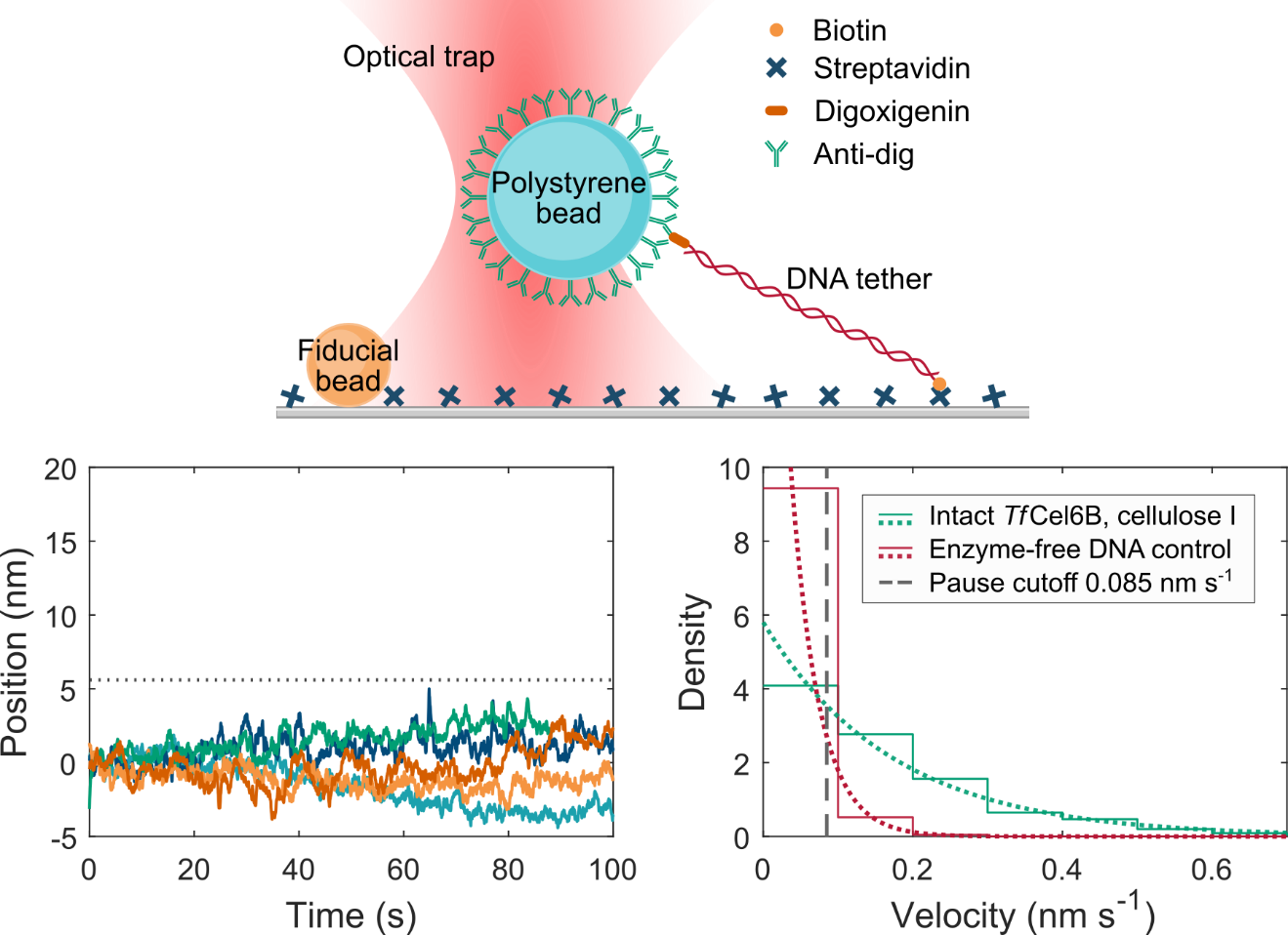
**

**Fig. S3. Enzyme-free controls.** Top: Cartoon representation of stationary tether control (not to scale). Bottom, left: Examples of drift-corrected records of stationary control tethers. Applied force ranged from 0.1 to 7.0 pN. The dotted line represents the mean forward run length of intact *Tf*Cel6B on CI (Figure 2B). Bottom, right: Probability density step histogram and exponential PDF (red) of observed “velocities” of stationary tethers after drift correction, compared to that of intact *Tf*Cel6B on CI (green). Control traces are segmented in triplicate in the same manner as motility traces, but velocities are not denoted as forward or backward. While some motion was unable to be corrected for using our drift tracking methods, the maximum observed motion was 0.22 nm s^-1^, with a mean of 0.037 ± 0.005 nm s^-1^ (N = 84, error 95% conf. int.) from the PDF fit. Integrating the PDF, 90% of all enzyme-free segments would be expected to have a velocity of less than 0.085 nm s^-1^ (dashed line) thus we consider segments moving faster than 0.085 nm s^-1^ to be enzyme-mediated motility.

**
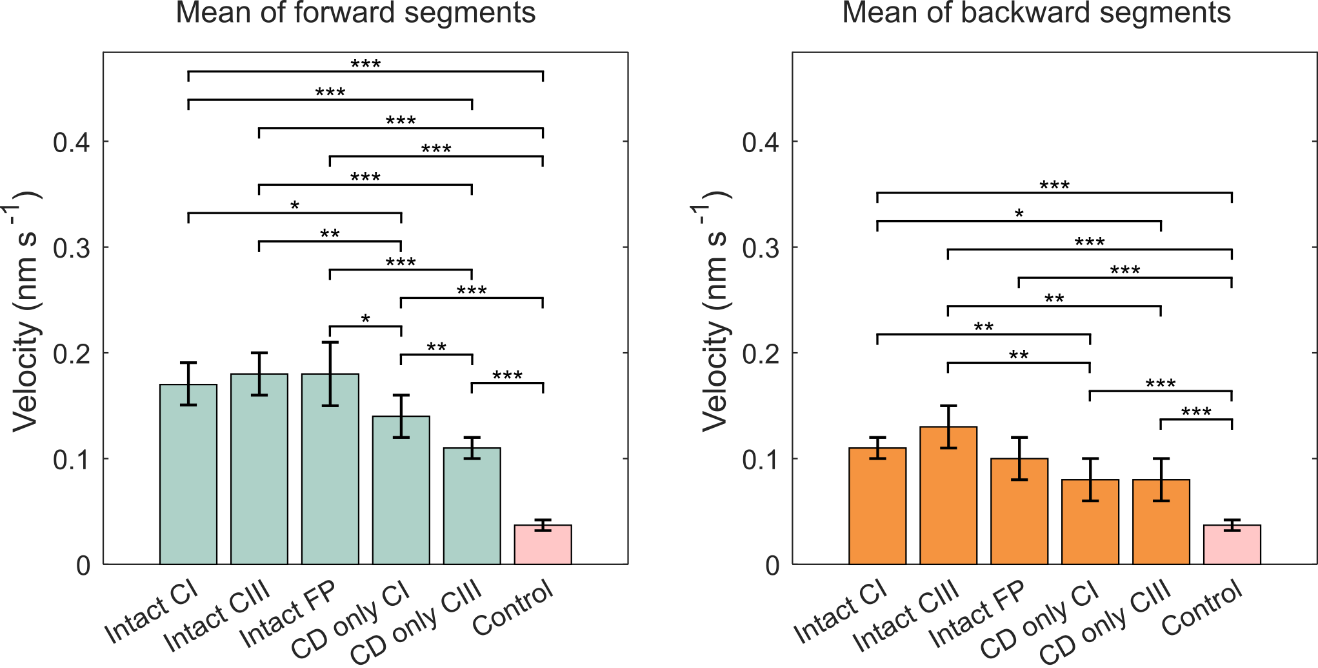
**

**Fig. S4.** **Comparison of mean segment velocities.** Bar plot of the time weighted average and 95% confidence intervals from exponential PDF fits of segment velocities shown in Figs. S1 and S3. Control segments were not split based on directionality thus the same mean is shown on both plots (pink). Datasets were compared using bootstrapping hypothesis testing, with H_0_: µ_1_ = µ_2_ and H_A_: µ_1_ ≠ µ_2_ (*, p<0.05; **, p<0.01; ***, p<0.0001). Any comparisons not marked were not significantly different. The control dataset was significantly different from all other datasets (p<0.0001).


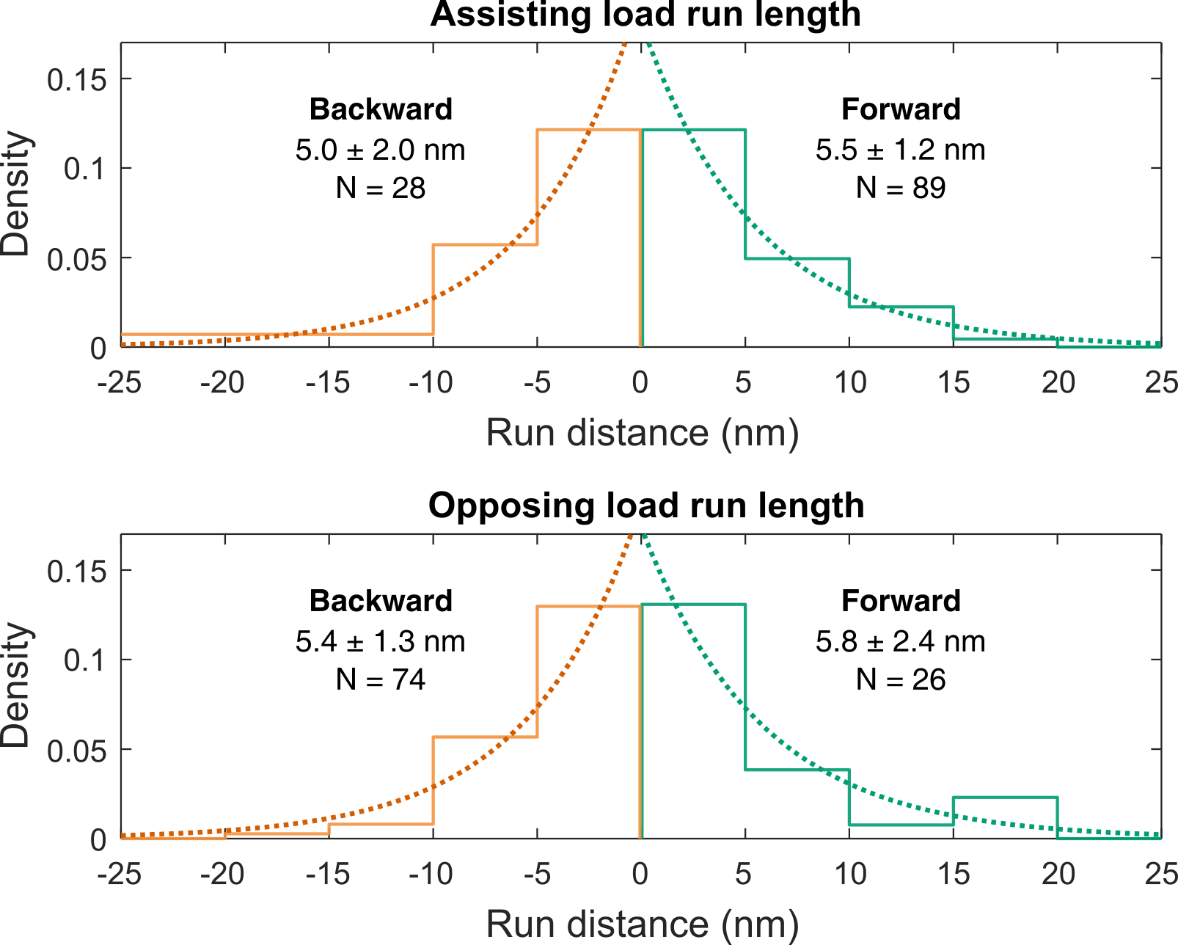


**Fig. S5. Run distance with respect to direction of force application.** Top: Run length distributions and PDFs of forward and backward runs in which the optical trap pulls in the direction of the run, with averages, 95% confidence intervals, and N from three replicate segmentations inset. Bottom: Run length distributions and PDFs in which the trap pulls opposite the run direction, with averages, 95% confidence intervals, and N from three replicate segmentations inset.

**
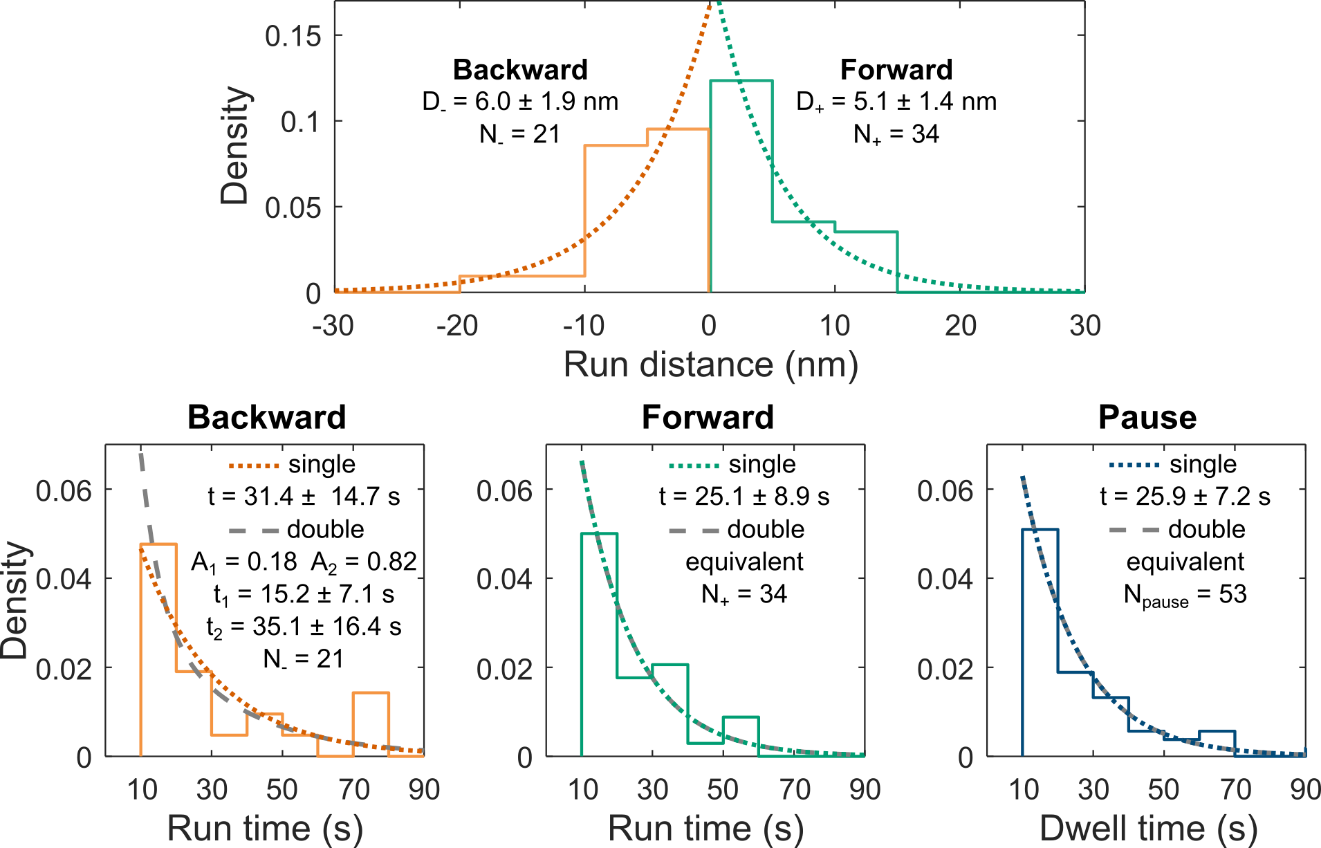
**

**Fig. S6. Run and pause distances and durations for CD only.** Top: run distance probability distributions overlaid with single exponential PDF fits for CD only on CI at 21°C. Bottom: run durations and pause dwell times overlaid with single and double exponential PDF fits. For all panels, inset are fit parameters ± 95% conf. intervals from PDF fits and N over three segmentation replicates. Double exponential PDF fits of forward run times and pause dwells were not able to parse out two populations, thus the fits are equivalent to the single exponential PDF.

**
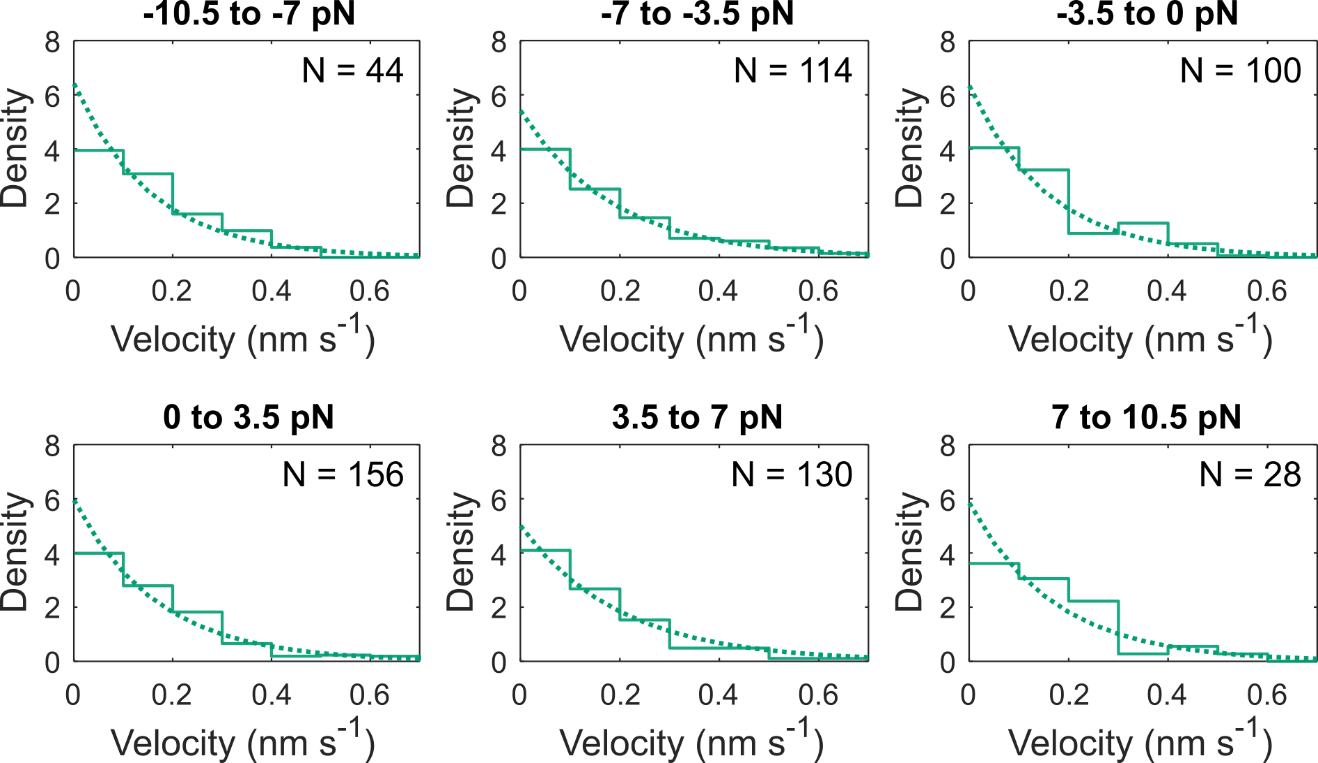
**

**Fig. S7. Distributions within force bins seen in Figure 4A.** The forward segments from all records of intact *Tf*Cel6B moving on cellulose I, cellulose III, or filter paper were combined and binned into 3.5 pN bins based on average force applied during the segment. The distribution within each bin, (green step histogram), was then fit to time weighted exponential PDFs (overlaid) to extract the mean velocities and 95% confidence intervals that are plotted in Figure 4A.


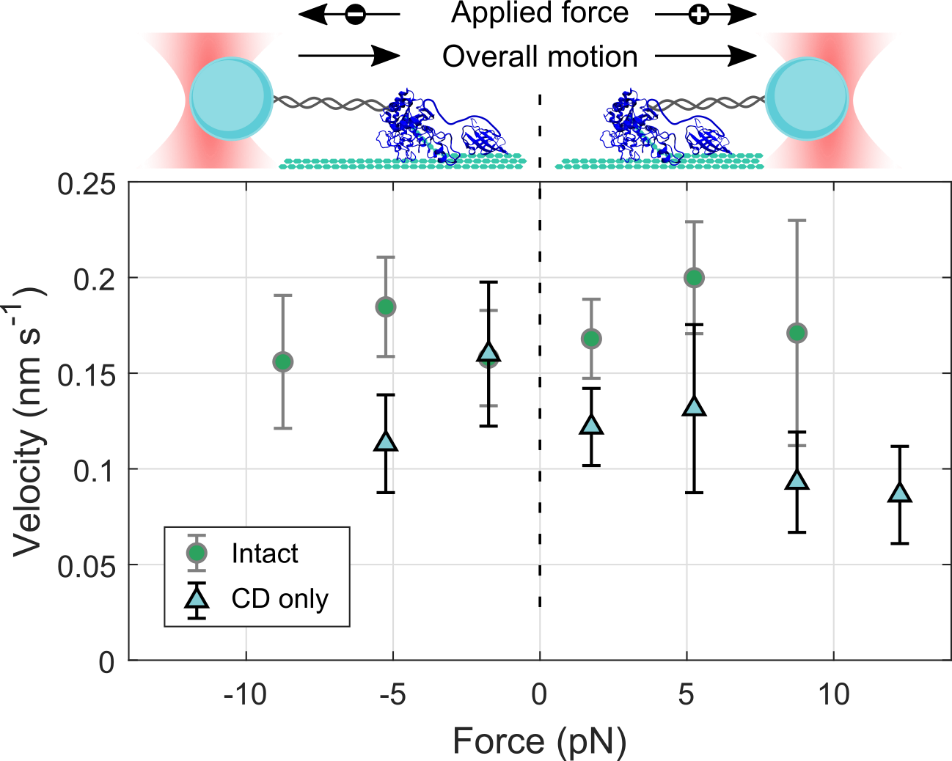


**Fig. S8. Impact of force on CD only velocity.** Blue triangles: CD only velocities with respect to applied force. Green circles: Intact velocities from Figure 4A. Negative force indicates opposing load geometry (see cartoon above), and positive force indicates assisting load. To maximize N, CD only segments collected on both cellulose I and cellulose III were combined and segmented in triplicate (N=248). Segments were binned by average force into 3.5 pN wide bins. Each point is the mean and 95% confidence interval calculated from fitting a PDF to the velocity distribution within each bin. The datapoint representing the 10.5 to 14 pN bin comes from a single record that sustained up to 14 pN of assisting load, but no CD only record sustained more than 7 pN of opposing load. (-7 to -3.5 pN: N=48; -3.5 to 0 pN: N=40; 0 to 3.5 pN: N=85; 3.5 to 7 pN: N=23; 7 to 10.5 pN: N=26; 10.5 to 14 pN: N=26).

**
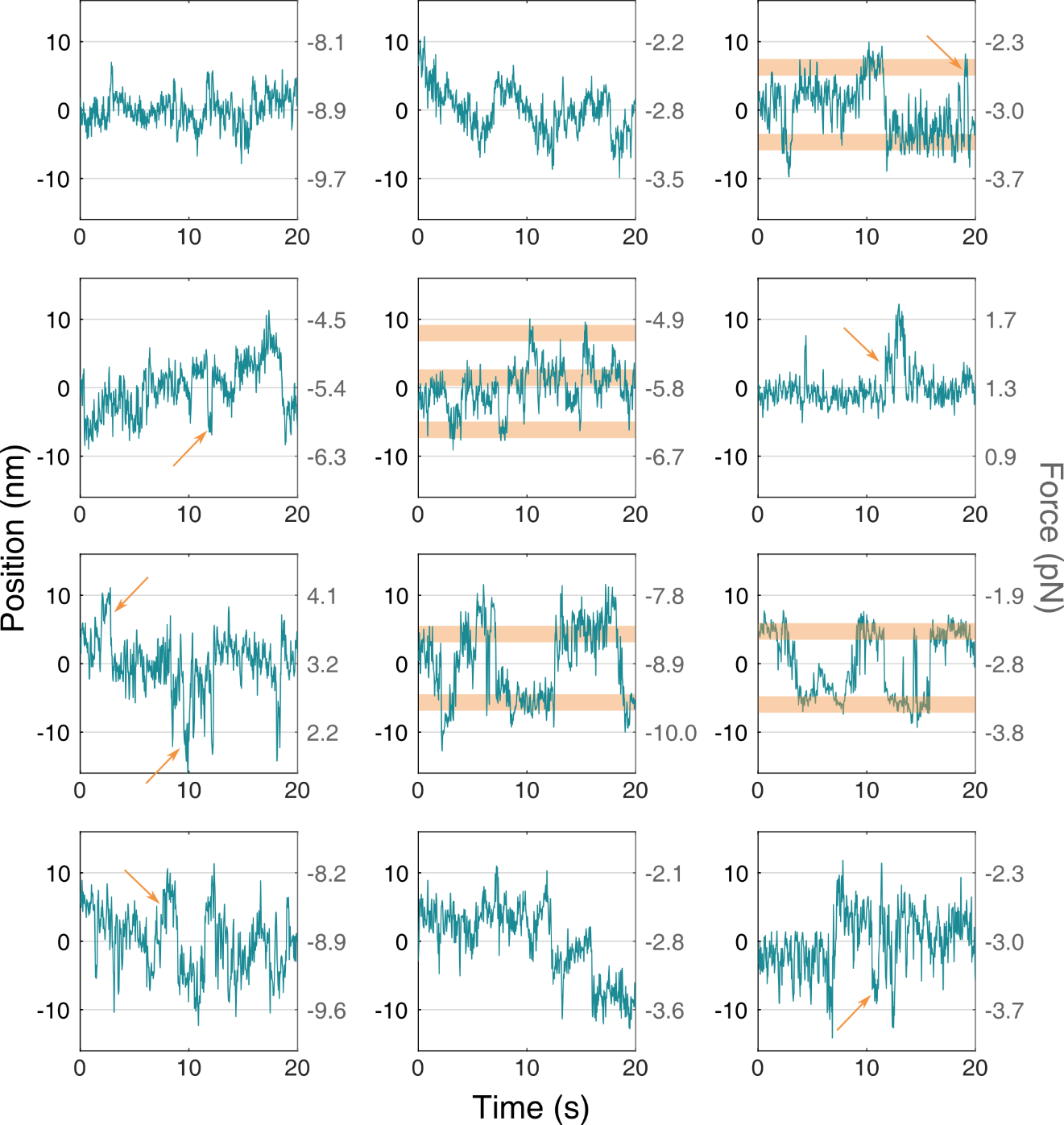
**

**Fig. S9.** **Examples of fast displacements in *Tf*Cel6B records.** A representative sample of fast, large displacements from all datasets collected at 21°C, centered at 0 nm for clarity. Right axis is applied force; positive force is assisting load (along the direction of overall trace progress) and negative is opposing load. Shaded regions indicate locations that the record appears to visit repeatedly. Arrows indicate examples of reversible “toggling,” or a fast position change followed by a return to the approximate starting position. Arrows and shading are not inclusive of all such events.

**
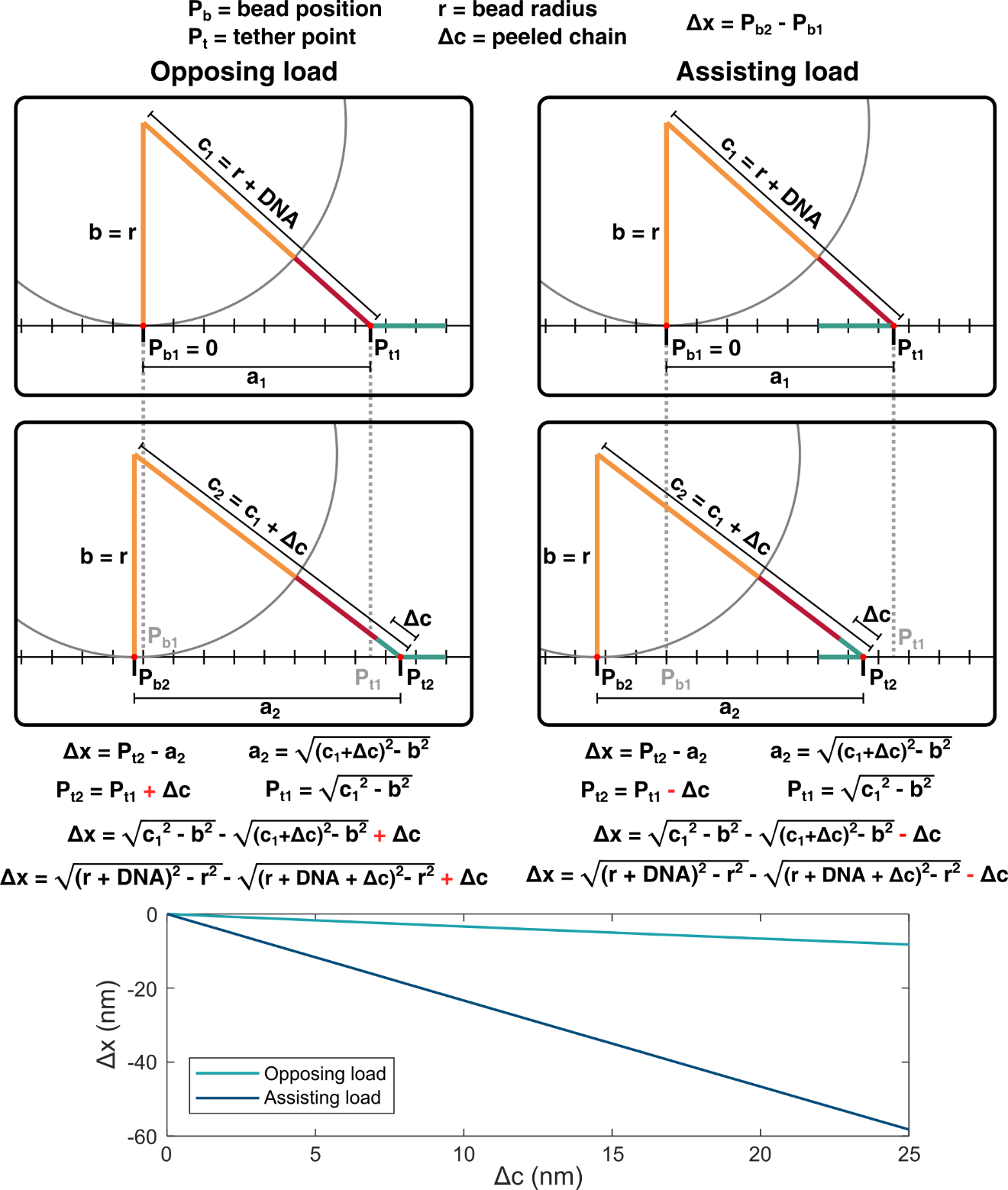
**

**Fig. S10. Direction of chain peeling causes disparities in bead motion.** Top: to-scale diagrams of chain peeling and the resulting equations for bead motion as a function of peeled chain length using the Pythagorean theorem. In the opposing load orientation, peeling up of a cellulose chain would only result in modest bead motion towards the trap center. In the assisting load orientation, the chain would be folded back, causing much larger changes in bead position. Graph (bottom) plots the length of peeled chain vs the expected bead motion based on a 1.36 µm bead and a 1,010 bp DNA tether assuming there is negligible distance between the bead and the surface.

**
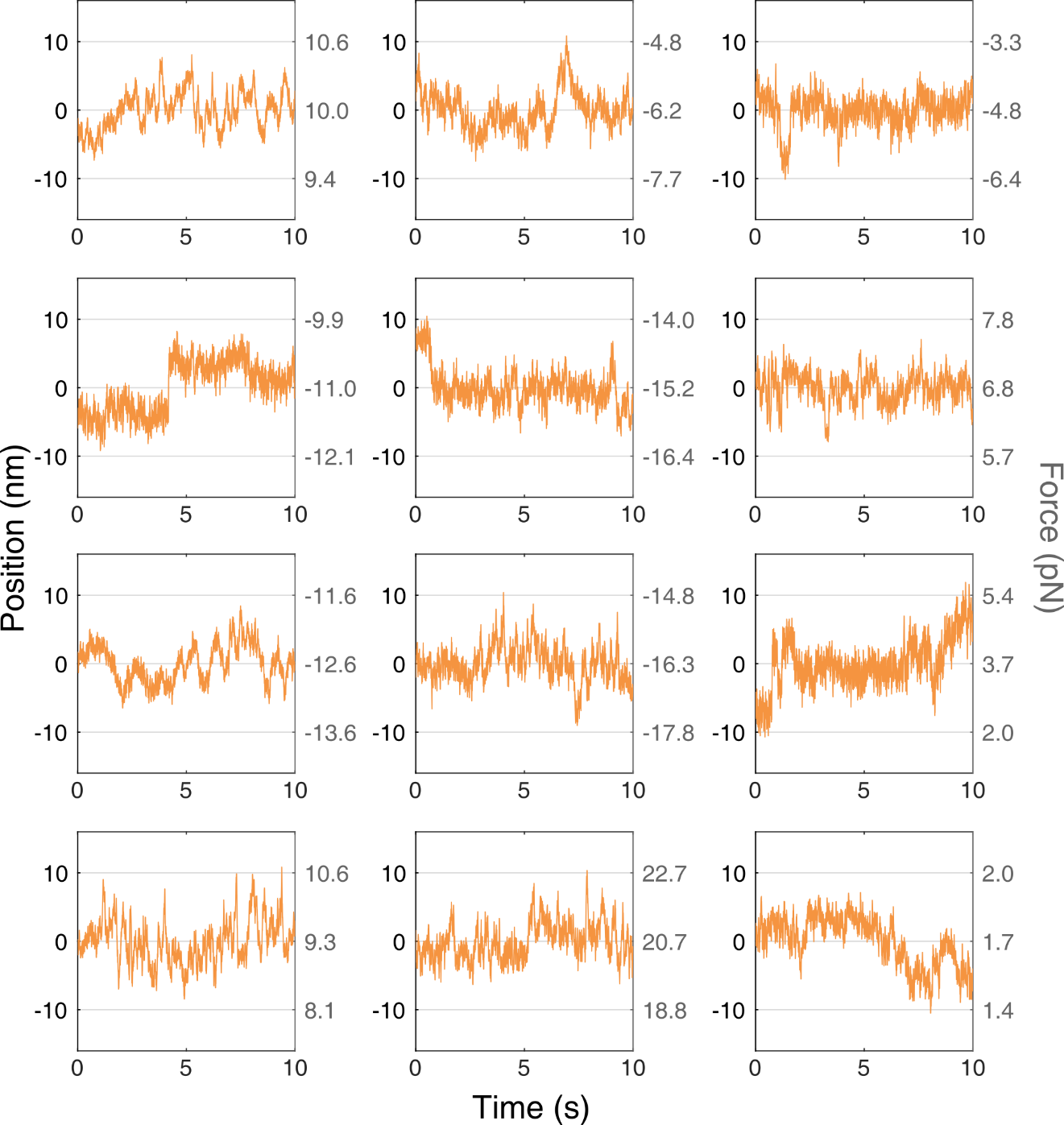
**

**Fig. S11. *Tr*Cel7A fast displacement examples.** Records collected by Brady et al., 2015 were reexamined in search of fast displacements similar to those shown in Fig. S9 [1]. Above records are a sampling of *Trichoderma reesei* Cel7A motility traces on filter paper at 21°C. Displacements >10 nm were more frequent for *Tf*Cel6B than *Tr*Cel7A, but motions of ~5 nm were common for both datasets. Traces are centered at 0 nm for clarity.

**Table S1. Summary of single-molecule motility record datasets.**

| Enzyme form | Substrate | Temp. (°C) | N (records) | Observation time (s) | Overall mean velocity  (nm s^-1^)* | Standard deviation (nm s^-1^)** |
| --- | --- | --- | --- | --- | --- | --- |
| Intact | Cellulose I | 21 | 22 | 3,413 | 0.05 | 0.10 |
| Intact | Cellulose III | 21 | 23 | 2,122 | 0.06 | 0.08 |
| Intact | Filter paper | 21 | 11 | 1,136 | 0.07 | 0.07 |
| CD only | Cellulose I | 21 | 16 | 1,293 | 0.08 | 0.18 |
| CD only | Cellulose III | 21 | 11 | 1,648 | 0.04 | 0.08 |
| Intact | Cellulose I | 27 | 4 | 194 | 0.13 | 0.19 |
| Intact | Cellulose I | 34 | 9 | 182 | 0.47 | 0.59 |

*Calculated by fitting a line to each record and taking the time-weighted average of all slopes

**Time-weighted

**Table S2. Mean velocities by segmentation iteration.**

| Conditions | Direction | Segmentation 1  (nm s^-1^ ± 95% conf. int) | Segmentation 2  (nm s^-1^ ± 95% conf. int) | Segmentation 3  (nm s^-1^ ± 95% conf. int) | Combined dataset  (nm s^-1^ ± 95% conf. int) |
| --- | --- | --- | --- | --- | --- |
| Intact  Cellulose I  21°C | Forward | 0.19 ± 0.03  N = 93 | 0.15 ± 0.02  N = 80 | 0.18 ± 0.03  N = 99 | 0.17 ± 0.02  N = 272 |
|  | Backward | 0.12 ± 0.02  N = 63 | 0.08 ± 0.01  N =47 | 0.13 ± 0.02  N = 65 | 0.11 ± 0.01  N = 175 |
| Intact  Cellulose III  21°C | Forward | 0.17 ± 0.03  N = 67 | 0.15 ± 0.02  N = 60 | 0.21 ± 0.04  N = 67 | 0.18 ± 0.02  N = 194 |
|  | Backward | 0.12 ± 0.03  N = 30 | 0.12 ± 0.03  N = 28 | 0.13 ± 0.03  N = 44 | 0.13 ± 0.02  N = 102 |
| Intact  Filter paper  21°C | Forward | 0.16 ± 0.04  N = 37 | 0.19 ± 0.04  N = 35 | 0.20 ± 0.05  N =34 | 0.18 ± 0.03  N = 106 |
|  | Backward | 0.09 ± 0.03  N = 19 | 0.12 ± 0.03  N = 22 | 0.08 ± 0.02  N =21 | 0.10 ± 0.02  N = 62 |
| CD only  Cellulose I  21°C | Forward | 0.12 ± 0.02  N = 36 | 0.15 ± 0.03  N = 36 | 0.14 ± 0.03  N =34 | 0.14 ± 0.02  N = 106 |
|  | Backward | 0.08 ± 0.02  N = 14 | 0.08 ± 0.02  N = 13 | 0.09 ± 0.03  N = 16 | 0.08 ± 0.02  N = 43 |
| CD only  Cellulose III  21°C | Forward | 0.10 ± 0.02  N = 54 | 0.10 ± 0.02  N = 47 | 0.12 ± 0.02  N =41 | 0.11 ± 0.01  N = 142 |
|  | Backward | 0.14 ± 0.04  N = 20 | 0.07 ± 0.02  N = 19 | 0.07 ± 0.02  N = 31 | 0.08 ± 0.02  N = 70 |
| Intact  Cellulose I  27°C | Forward | 0.22 ± 0.09  N = 5 | 0.34 ± 0.15  N = 4 | 0.42 ± 0.19  N = 8 | 0.31 ± 0.09  N = 17 |
|  | Backward | 0.28 ± 0.16  N = 1 | 0.23 ± 0.13  N = 3 | 0.18 ± 0.09  N = 5 | 0.22 ± 0.08  N = 9 |
| Intact  Cellulose I  34°C | Forward | 0.73 ± 0.31  N = 8 | 0.56 ± 0.24  N = 8 | 0.61 ± 0.25  N = 10 | 0.63 ± 0.18  N = 26 |
|  | Backward | N = 0 | N = 0 | N = 0 | N = 0 |
| Control  21°C | N/A | 0.036 ± 0.007  N = 25 | 0.034 ± 0.007  N = 26 | 0.040 ± 0.008  N=33 | 0.037 ± 0.005  N = 84 |
| Control  34°C | N/A | 0.17 ± 0.06  N = 18 | 0.23 ± 0.09  N = 16 | 0.25 ± 0.10  N = 22 | 0.22 ± 0.05  N = 56 |

**Table S3. Results from two-way ANOVA comparing bulk hydrolysis rates from Figure 3B.**

| **Source of Variation** | **Degrees of Freedom** | **F-statistic** | **P-value** |
| --- | --- | --- | --- |
| Enzyme form | 1 | 496.1 | 3.95x10^-11^ |
| Substrate | 2 | 9.9 | 2.93x10^-3^ |
| Interaction | 2 | 4.5 | 0.03 |

**Table S4. Post-hoc testing of bulk hydrolysis rates from Figure 3B using Tukey’s Honestly Significant Data test.**

|  | **Intact, CIII** | **Intact, FP** | **CD only, CI** | **CD only, CIII** | **CD only, FP** |
| --- | --- | --- | --- | --- | --- |
| Intact, CI | * | X | *** | *** | *** |
| Intact, CIII |  | ** | *** | *** | *** |
| Intact, FP |  |  | *** | *** | *** |
| CD, CI |  |  |  | X | X |
| CD, CIII |  |  |  |  | X |

X: Means are not significantly different

*: Means are unequal at α=0.05

**: Means are unequal at α=0.005

***: Means are unequal at α=0.001

**References**

1. Brady SK, Sreelatha S, Feng Y, Chundawat SPS, Lang MJ. Cellobiohydrolase 1 from *Trichoderma reesei* degrades cellulose in single cellobiose steps. Nat Commun. 2015;6:10149.
